# Exosomal Lnc NEAT1 from endothelial cells promote bone regeneration by regulating macrophage polarization via DDX3X/NLRP3 axis

**DOI:** 10.1186/s12951-023-01855-w

**Published:** 2023-03-20

**Authors:** Yuxuan Chen, Yuanhao Wu, Linlin Guo, Shijie Yuan, Jiaming Sun, Kangcheng Zhao, Jiecong Wang, Ran An

**Affiliations:** 1 Department of Plastic Surgery, Union Hospital, Tongji Medical College, Huazhong University of Science and Technology, Wuhan, 430022 China; 2 Department of Vascular Surgery, Union Hospital, Tongji Medical College, Huazhong University of Science and Technology, Wuhan, 430022 China; 3 Department of Immunology, School of Basic Medicine, Tongji Medical College, Huazhong University of Science and Technology, Wuhan, 430022 China; 4 Department of Orthopaedics, Union Hospital, Tongji Medical College, Huazhong University of Science and Technology, Wuhan, 430022 China

**Keywords:** Exosome, Macrophage polarization, NEAT1, Osteogenesis, Inflammation

## Abstract

**Background:**

Bone regeneration is a complex procedure that involves an interaction between osteogenesis and inflammation. Macrophages in the microenvironment are instrumental in bone metabolism. Amount evidence have revealed that exosomes transmitting lncRNA is crucial nanocarriers for cellular interactions in various biotic procedures, especially, osteogenesis. However, the underlying mechanisms of the regulatory relationship between the exosomes and macrophages are awaiting clarification. In the present time study, we aimed to explore the roles of human umbilical vein endothelial cells (HUVECs)-derived exosomes carrying nuclear enrichment enriched transcript 1 (NEAT1) in the osteogenesis mediated by M2 polarized macrophages and elucidate the underlying mechanisms.

**Results:**

We demonstrated HUVECs-derived exosomes expressing NEAT1 significantly enhanced M2 polarization and attenuated LPS-induced inflammation in vitro*.* Besides, the conditioned medium from macrophages induced by the exosomes indirectly facilitated the migration and osteogenic differentiation of bone marrow-derived mesenchymal stem cells (BMSCs). Mechanically, Exos carrying NEAT1 decreased remarkably both expression of dead-box helicase 3X-linked (DDX3X) and nod-like receptor protein 3 (NLRP3). The level of NLRP3 protein increased significantly after RAW264.7 cells transfected with DDX3X overexpression plasmid. Additionally, the knockdown of NEAT1 in exosomes partially counteracted the aforementioned effect of Exos. The results of air pouch rat model demonstrated that HUVECs-derived exosomes increased anti-inflammatory cytokines (IL-10) and decreased pro-inflammatory cytokines (IL-1β and IL-6) significantly in vivo, contributing to amelioration of LPS-induced inflammation. Afterwards, we further confirmed that the HUVECs-derived exosomes encapsulated in alginate/gelatin methacrylate (GelMA) interpenetrating polymer network (IPN) hydrogels could promote the bone regeneration, facilitate the angiogenesis, increase the infiltration of M2 polarized macrophages as well as decrease NLRP3 expression in the rat calvarial defect model.

**Conclusions:**

HUVECs-derived exosomes enable transmitting NEAT1 to alleviate inflammation by inducing M2 polarization of macrophages through DDX3X/NLRP3 regulatory axis, which finally contributes to osteogenesis with the aid of alginate/GelMA IPN hydrogels in vivo. Thus, our study provides insights in bone healing with the aid of HUVECs-derived exosomes-encapsulated composite hydrogels, which exhibited potential towards the use of bone tissue engineering in the foreseeable future.

**Supplementary Information:**

The online version contains supplementary material available at 10.1186/s12951-023-01855-w.

## Introduction

Cranial reconstruction is as such among the most demanding issues in craniomaxillofacial surgery for skull defect, mainly in brain tumor operations, traumatic injuries, craniotomies, and congenital cranial anomalies [1]. Current therapies for repairing skull defects normally use a cranial implant to accurately replace the absent cranial bone, include autografts, allografts and xenografts, metal biomaterials and macromolecular biomaterials [2, 3]. In order to best match the apertures on the cranium, the customized material and plastic bone replacements need to correspond accurately to the morphology of defect, as well as facilitate the bone regeneration.

Recent studies have indicated that the polarization state of macrophage was instrumental in bone healing [4]. Macrophages play critical roles in removing the tissue debris and secreting different signaling macromolecules to enlist the progenitors. Depletion of macrophages has been reported to result in an impaired and delayed osseous repair in fracture models of murine, indicating that the vital contributions of macrophages to bone regeneration [5, 6]. Macrophages are mainly classified into M1 and M2 subgroups, decreasing the M1 phenotype and increasing the M2 phenotype could remarkably enhanced osteogenesis and vascularization, furthermore, leading to bone healing [7]. Pioneer works have illustrated the ability of mesenchymal stem cells (MSCs)-derived exosomes to promote differentiation of macrophages into the M2 subtype, thereby reducing inflammatory responses and promoting tissue repair [8]. Compelling evidence demonstrated that M2 macrophages have been gradually recognized as a positive regulator of bone formation [9]. Nevertheless, the fundamental mechanisms associated with macrophages that manipulate the fate of BMSCs remain unclear.

Exosomes are extracellular vesicles rich in DNA, RNA, lipids, and proteins [10]. With the development of exosome-based therapeutics, exosomes act as mediators in cell–cell communications and are able to re-program recipient cells according to the parent cell's acculturation environment [11, 12]. In addition, exosome-based cell-free therapy is gaining attention due to effectively avoiding the risks of low survival rate, strong immune rejection, and high tumorigenicity of mutations caused by the direct use of cells [13, 14]. Human umbilical vein endothelial cells (HUVECs) derived exosomes are effective for promoting diabetic wound healing, reducing reperfusion damage, and protecting nerve cells against ischemia/reperfusion injury [17–19]. Inspired by these studies, our previous study has confirmed that the exosomes derived from HUVECs significantly improved the angiogenesis ability of endothelial progenitor cells and increased the survival area of the flap in vivo through nuclear enrichment enriched transcript 1 (NEAT1)/Wnt/β-catenin signal pathway [15]. Such suggestions indicate that HUVECs-derived exosomes may serve as a novel therapeutic tool for tissue repair, such as bone regeneration. Given the potential benefits of HUVECs-derived exosomes, therefore, it is urgent to find a way to investigate the role of HUVECs-derived exosomes on bone regeneration in this study.

To address this, long noncoding RNAs (lncRNAs) could be loaded within exosomes to regulate gene expression in host cell via cell communication [16]. LncRNA NEAT1 is a classic lncRNA which resides in paraspeckles [17] and can act as an inflammatory meditor [18]. Of note, NEAT1 could also activate NLR family CARD domain containing 4 (NLRC4) and nod-like receptor protein 3 (NLRP3) inflammasome, and stabilize the caspase-1 to promote IL-1β production and pyroptosis [19–21]. However, several studies have also illustrated that NEAT1 could not only ameliorate LPS-induced inflammation, but inhibit the activation of NLRP3 inflammasome by interacting with specific pathways and miRNAs, finally leading to M2 polarization [20, 22]. Dead-box helicase 3X-linked (DDX3X) is essential for assembly of NLRP3 inflammasomes and stress granules due to its binding action to NLRP3 [23]. DDX3X deletion could alleviate cardiomyocyte pyroptosis induced by LPS via inhibiting NLRP3 inflammasome activation [24]. Abundant evidence has reported that the axis of DDX3X-NLRP3-mediated pyroptosis could be regulated by several factors, such as AKT, aluminum, TLR4 and so on [25–27]. Considering the undefined role of NEAT1 in immunity, works need to be done to decipher whether NEAT1 could regulate NLRP3 inflammasome activation mediated through DDX3X for macrophage polarization, suppression of chronic inflammation and bone regeneration.

In vivo*,* unloaded exosome-based therapeutics still face challenges, due to the short half-life and fast removal rate, especially applied in bone repair, which is a long-term and complex multiple process [28]. Therefore, it is critical to select a suitable carrier to carry exosomes to maintain the function of exosomes and achieve sustained release at the target site. Here, we report on an alginate/GelMA IPN hydrogel to deliver bioactive exosomes and prefabricated in a mold to match the defective region to promote osseous restoration.

In our work, we aimed to investigate the effect of exosomes derived from HUVECs associated with DDX3X/NLRP3 axis on osteogenesis. Our study revealed that NEAT1 in HUVECs-derived exosomes has dramatic effects on the regulation of macrophage plasticity to resolve chronic inflammation, promote the osteogenic function of BMSCs and enhance in vivo osteogenesis, via suppressing NLRP3 inflammasome activation regulated by DDX3X. We propose this study may provide potential insight for the therapeutic application of exosomal lncRNAs in osteogenesis.

## Results

### Characterization of exosomes

**Fig. 1:**
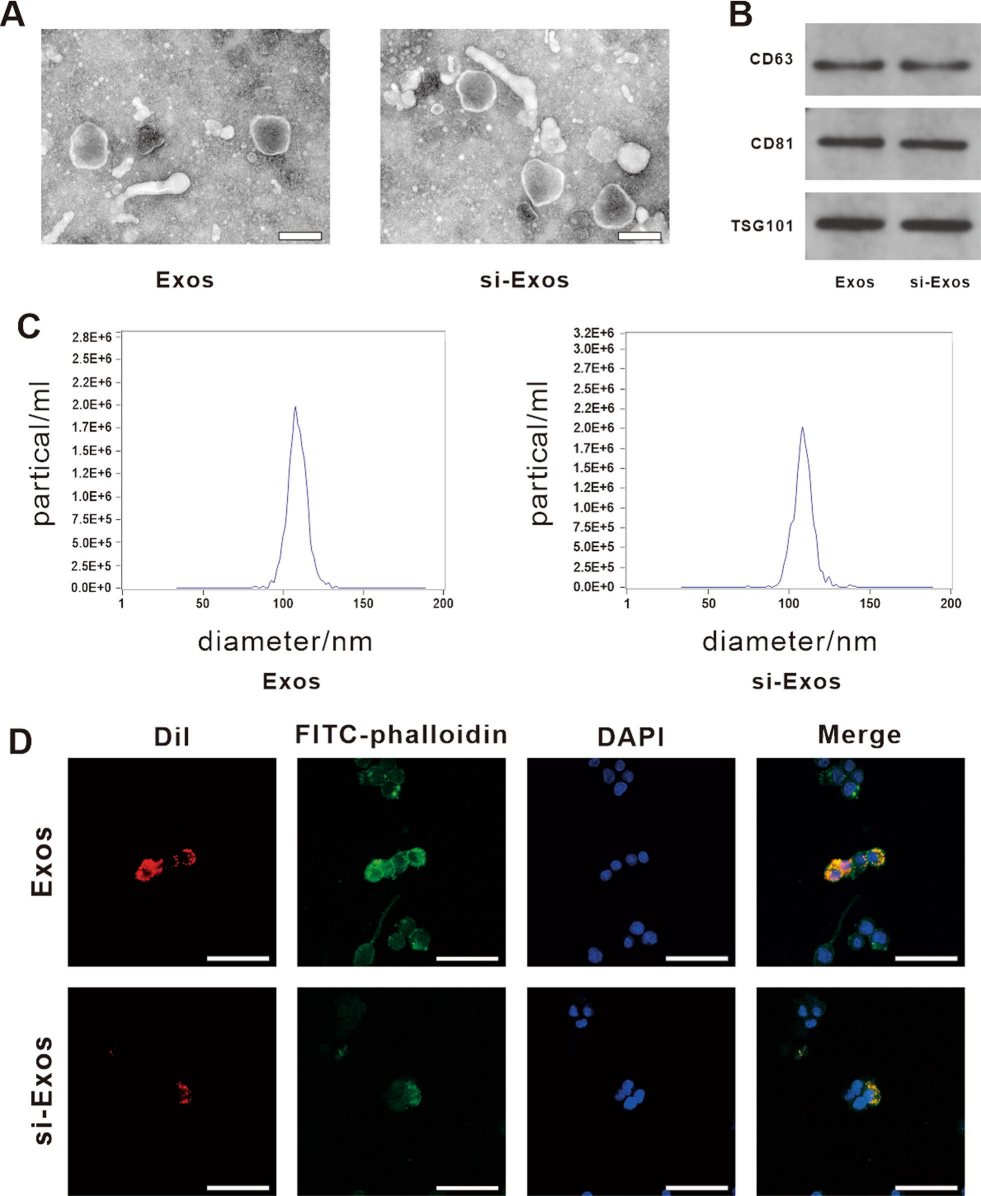
Characterization and internalization of HUVECs-derived Exos by RAW264.7. **A** Morphology of Exos and si-Exos identified by TEM. Scale bar = 200 nm. **B** Western blot analysis of the specific markers of exosomes, including CD63, CD81 and TSG101. **C** The particle size distribution and particle concentration of Exos and si-Exos detected by NTA. **D** The uptake of Exos and si-Exos by RAW264.7 cells. FITC-phalloidin (green) and DAPI (blue) were used to stain the cytoskeleton and nucleus of RAW264.7, respectively. Exosomes were labeled with Dil (red). Scale bar = 50 μm

Regarding the identification of exosomes, we performed nanoparticle tracking analysis (NTA), western blotting and TEM. The exosomes appeared as typical cup-shaped morphology by TEM (Fig. 1A). Western blotting analysis revealed the positive expression of exosomal specific markers CD63, CD81 and TSG101 in the isolated particles with no significant difference between the two groups (Fig. 1B). In addition, NTA results showed that the size distribution of exosomes from HUVECs (Exos) and HUVECs transfected with NEAT1 siRNA (si-Exos) displayed similar peak diameters of around 105 nm (Exos: 103.6 ± 44.4 nm, si-Exos: 108.5 ± 37.5 nm), and both the diameters of exosomes in the range of 50–150 nm was > 99% (Fig. 1C). As shown in Fig. 1D, the red fluorescence of Dil-labeled exosomes were clearly observed in the cytoplasm of RAW264.7 around the nucleus indicating the two kinds of exosomes could display a cellular transmission activity similarly which was not affected by parental NEAT1 depletion. Quantitative Real-time Polymerase Chain Reaction (qRT-PCR) data showed a significantly decrease in NEAT1 expression in the secreted exosomes following the transfection of the NEAT1 inhibitor, compared with the expression level in non-treated exosomes (Additional file 1: Fig. S1).

### Characterization of exosomes embedded with hydrogel

**Fig. 2:**
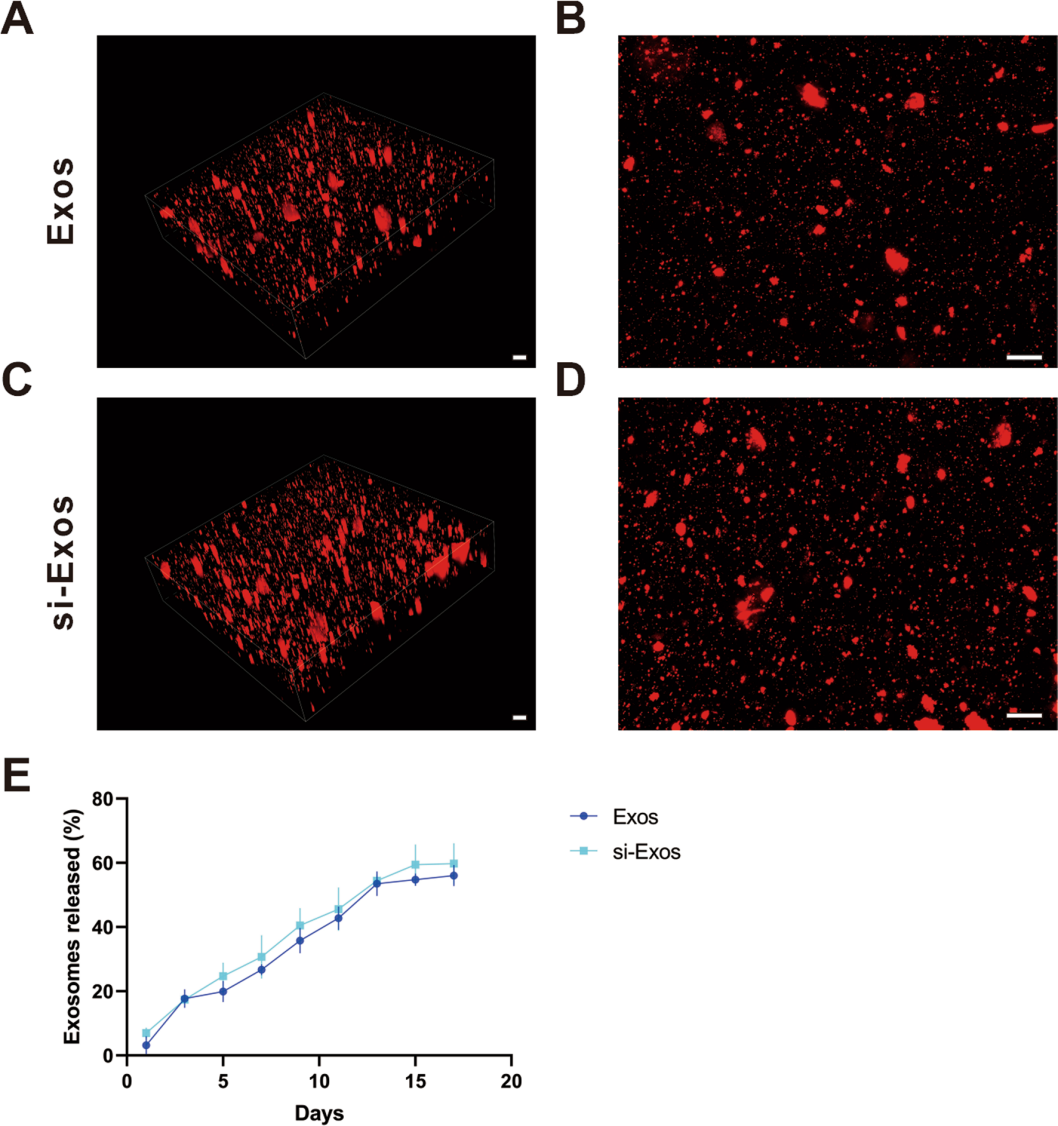
Exosome retention ability of alginate/GelMA IPN hydrogel. **A**/**C** 3D image of Dil-labeled Exos/si-Exos incorporated in alginate/GelMA IPN hydrogel. **B**/**D** overlapping image of **A**/**C**. **E** Release curves of Exos and si-Exo from composite hydrogels. Scale bar = 100 μm

To explore the effects of the exosomes in vivo, the Exos or si-Exos were embedded with alginate/GelMA IPN hydrogels to establish composites. According to the 3D reconstruction images of exosomes encapsulated in hydrogels (Fig. 2A–D), amount of red fluorescence Dil-labeled exosomes were homogeneously distributed in the hydrogels. Figure 2E indicated the composite hydrogels had a continuously slow and controlled release effect on exosomes during the monitoring span. The release curves of two different kinds of exosomes in hydrogel showed similar trends and no significant differences were observed at all time points between the two groups. Notably, approximately 50% of the exosomes were still remained inside the hydrogels after 15 days.

### HUVECs derived exosomes attenuated inflammation by enhancing M2 polarization in vitro

To determine the macrophage polarization state under stimulation with Exos and si-Exos after treated with LPS, we tested M1 and M2 polarization markers by flow cytometry, enzyme-linked immunosorbent assay (ELISA) and qRT-PCR. CD86, IL-1β and IL-6 represent the inflammatory state of M1 polarization, while CD206, IL-10 and Arg1 are the markers of anti-inflammatory state of M2 polarization.

**Fig. 3:**
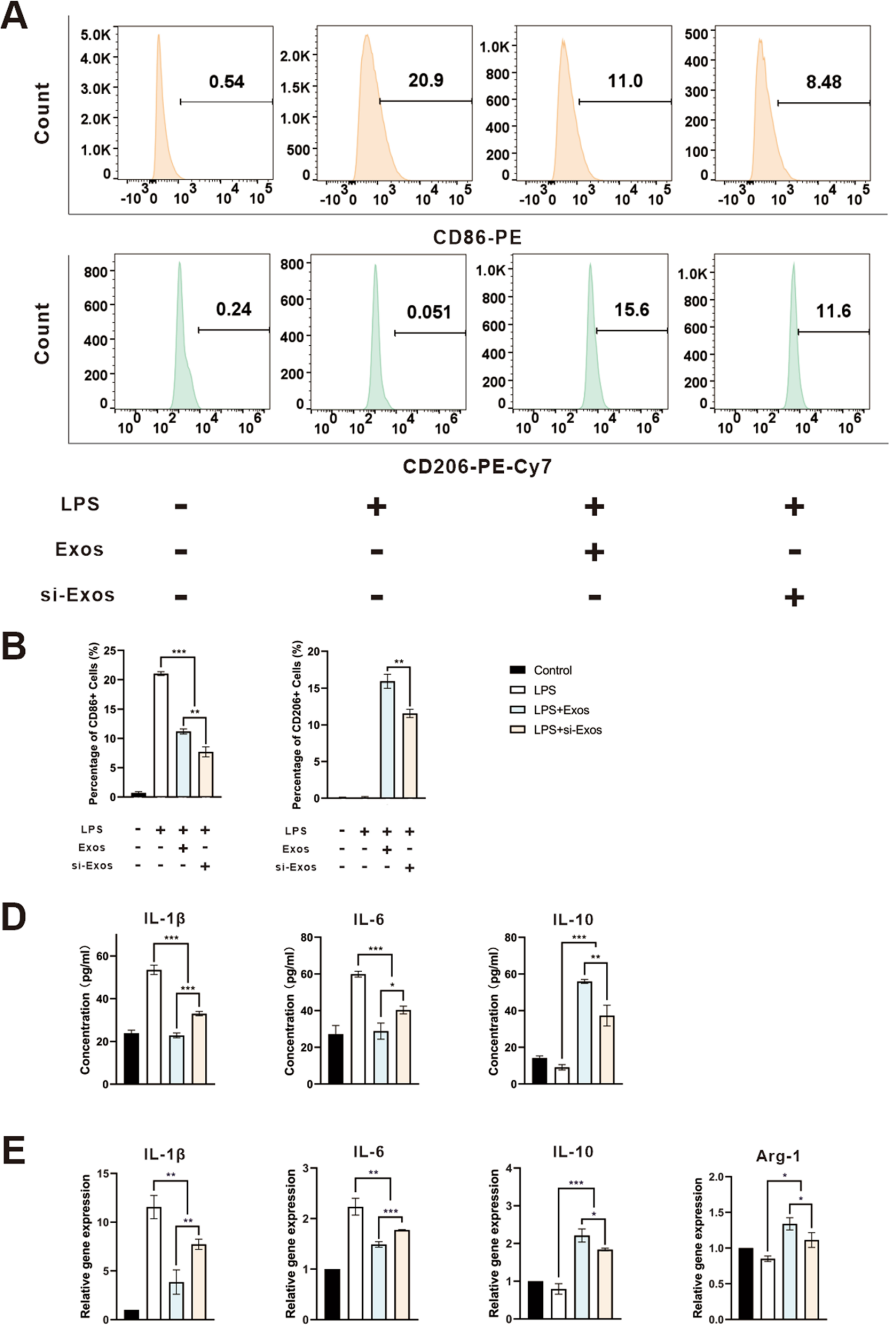
HUVECs-derived Exos attenuated inflammation by enhancing M2 polarization in vitro*.* **A** Representative images of the percentage of CD86 and CD206 positive cells detected by flow cytometry analysis. **B** Quantification of flow cytometry analysis of the percentage of CD86 and CD206 positive cells. **C** The concentrations of IL-1β, IL-6 and IL-10 of the supernatants detected by ELISA. **D** The relative gene expression of IL-1β, IL-6, IL-10 and Arg1 detected by qRT-PCR. * P < 0.05, ** P < 0.01, *** P < *0.001*

By flow cytometry, CD86 expression was significantly increased in LPS group, but markedly decreased after treated with the two kinds of exosomes. In contrast, the expression of M2 macrophage marker (CD206) significantly elevated with the treatment of the exosomes to varying degrees. Meanwhile, the knockdown of NEAT1 resulted in lower proportions of M2 macrophages than did normal HUVECs-derived exosomes (Fig. 3A/B, *P* < *0.01*). Next, by ELISA, IL-1β as well as IL-6 secretion were significantly decreased, while IL-10 secretion was markedly increased in the groups treated with Exos and si-Exos, especially treated with Exos, compared to the control group (Fig. 3C, P < *0.05*).

Furthermore, qRT-PCR results showed that the expression levels of IL-1β and IL-6 was significantly decreased, while the expression of IL-10 and Arg-1 was significantly increased in Exos and si-Exos groups, with the highest enhancement in Exos group. (Fig. 3D, P < *0.05*).

### HUVECs-derived exosomes promoted osteogenic differentiation and migration of BMSCs

To explore whether the exosomes could promote the osteointegration by enhancing M2 polarization, the supernatant from RAW264.7 with different treatments (PBS, LPS, LPS + Exos, LPS + si-Exos) as conditioned medium (CM) were collected.

**Fig. 4:**
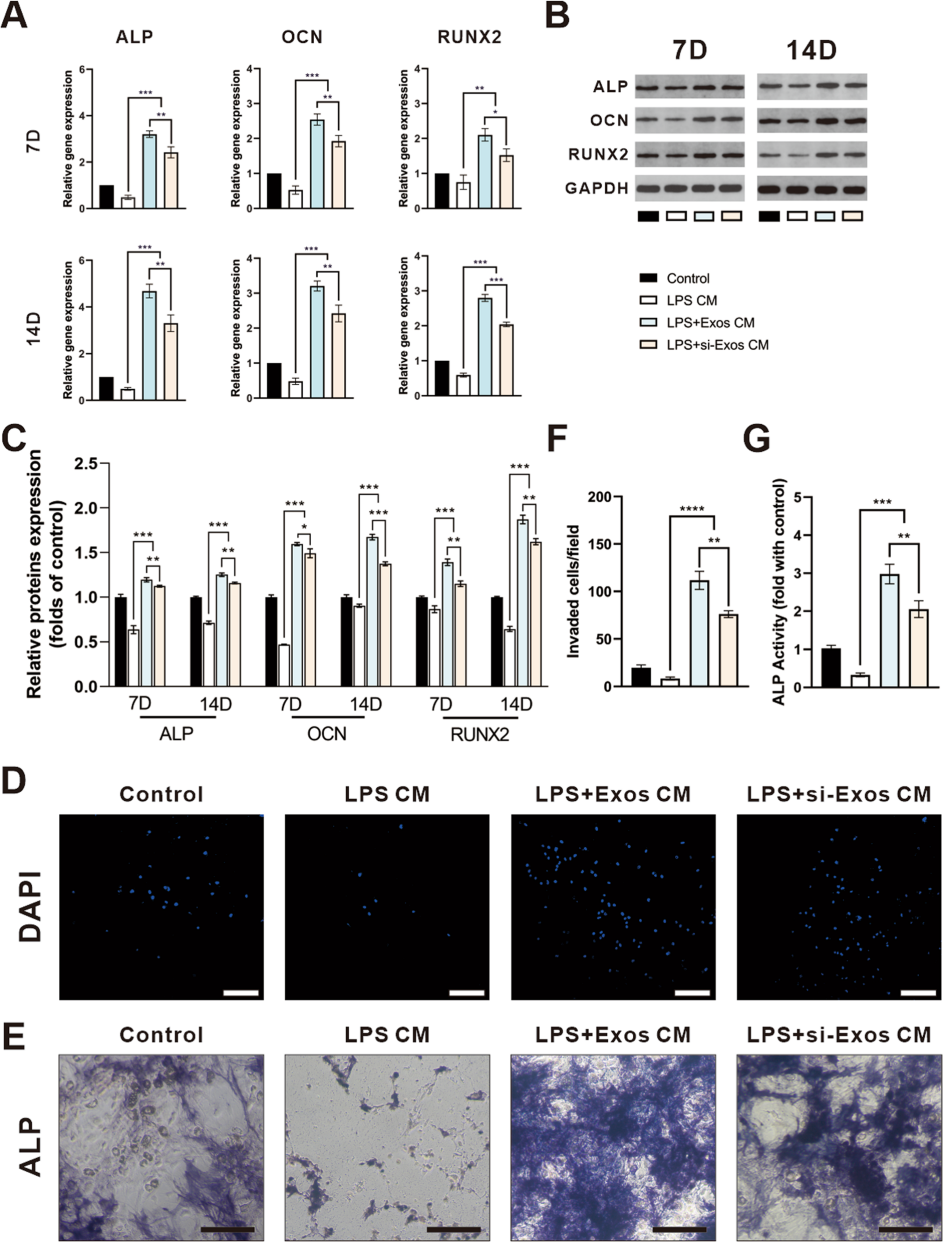
Conditional medium from macrophages treated by the exosomes promoted migration and osteogenic differentiation of BMSCs. **A** qRT-PCR analysis for mRNA expressions of ALP, OCN and RUNX2 on day 7 and day 14. **B** Western blot analysis and quantification **C** of protein levels of ALP, OCN and RUNX2 on day 7 and day 14. **D** Representative images of transwell assay and quantification (**F**) of cell migration. Representative images (**E**) and quantification (**G**) of ALP staining after 7 days of osteogenic induction. Scale bar = 200 μm. * P < 0.05, ** P < 0.01, *** P < *0.001*

By RT-PCR assay, the mRNA levels of osteogenic genes, alkaline phosphatase (ALP), OCN and RUNX2 of BMSCs significantly elevated in either LPS + Exos CM or LPS + si-Exos CM from days 7 -14 days compared to the other groups (Fig. 4A, P < *0.05*). Likewise, RUNX2, OCN and ALP were upregulated dramatically through a western blotting assay (Fig. 4B/C, *P* < *0.05*). Accordantly, after knockdown of NEAT1, the positive effect of HUVECs-derived exosomes on osteogenesis of BMSCs was partially attenuated.

To further elucidate the effect of NEAT1 on the migration capacity of BMSCs, transwell assay was performed. As shown in Fig. 4D/F, LPS + Exos CM and LPS + si-Exos CM attracted more MSCs to the lower chamber than did LPS CM group (*P* < *0.01*). However, conditioned medium derived from unstimulated macrophages had no apparent influence on the migratory ability of BMSCs. Meanwhile, there was a significance difference from the LPS + Exos CM versus LPS + si-Exos CM (*P* < *0.01*), indicating a stronger transmigrated capability of the HUVECs-derived exosomes without NEAT1 inhibition.

Similarly, ALP activity was significantly increased in the LPS + Exos CM and the LPS + si-Exos CM group compared to the other two groups, which was highest in the LPS + Exos CM group (Fig. 4E/G, *P* < *0.01*).

### Exosomal NEAT1 inhibited LPS-induced inflammation via the DDX3X/NLRP3 pathway

**Fig. 5:**
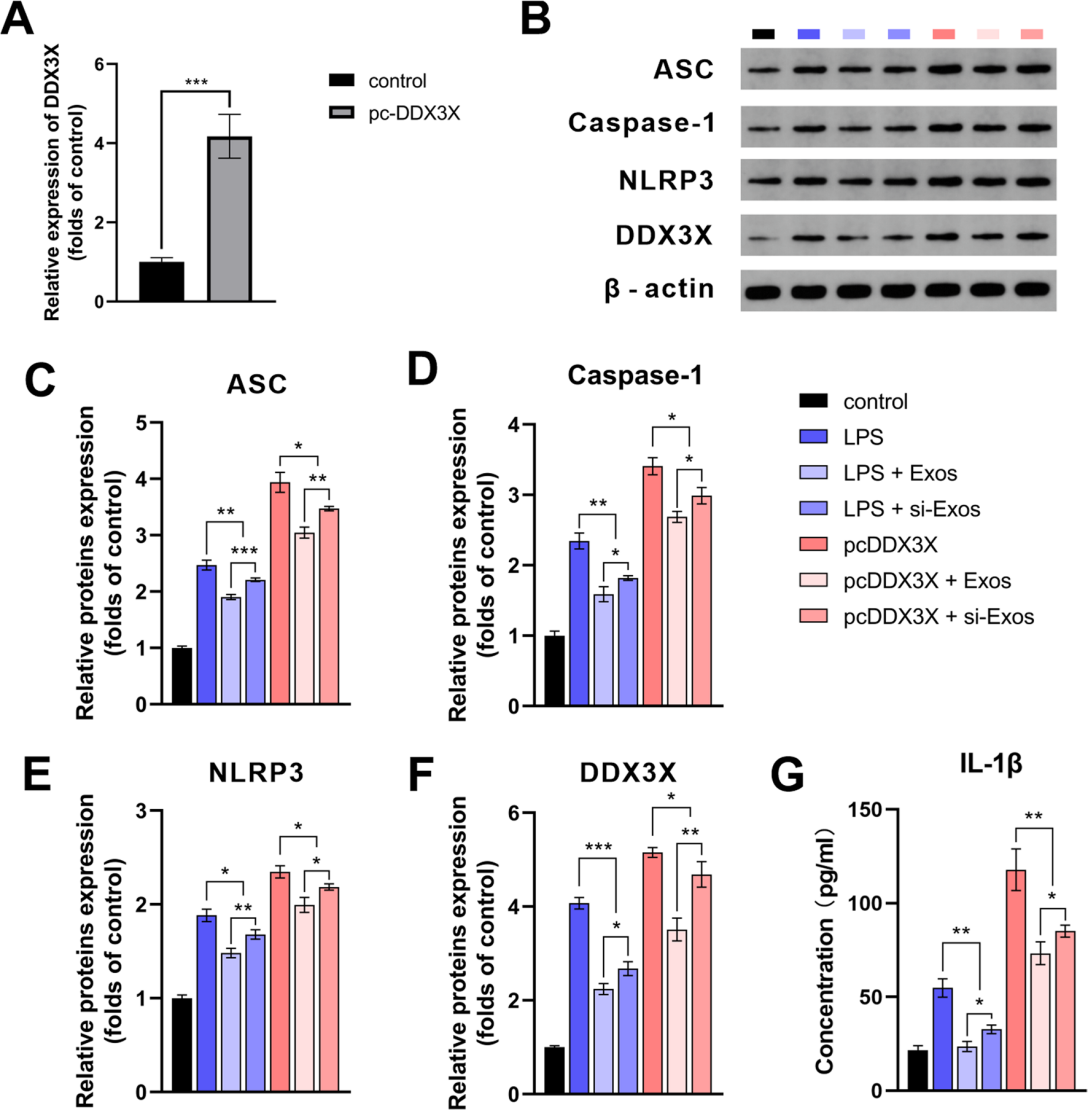
Lnc NEAT1 inhibited NLRP3 inflammasome activation by targeting DDX3X. **A** qRT-PCR analysis for mRNA level of DDX3X. **B**–**F** Western blot analysis and quantification of protein levels of ASC, Caspase-1, NLRP3 and DDX3X. **G** ELISA analysis for the concentrations of IL-1β of the supernatants. * P < 0.05, ** P < 0.01, *** P < *0.001*

To investigate the underlying mechanisms of NEAT1 mediated the HUVEC derived exosomes regulation of osteogenesis, RAW264.7 cells were successfully transfected with DDX3X overexpression plasmid. The transfection efficiency was verified by qRT-PCR (Fig. 5A).

Immunoblotting demonstrated that the expression levels of ASC, Caspase-1 and NLRP3 were found to be significantly increased following LPS stimulation and DDX3X overexpression. Besides, the expression of NLRP3 increased following the application of NEAT1 inhibited exosomes. NEAT1-shRNA transfection increased the LPS-induced increase in protein expression of ASC, Caspase-1, NLRP3 and DDX3X. The changes in the expression of NLRP3 in each group were in accordance with those of DDX3X indicating the inhibitory effect of NEAT1 on NLRP3 was dependent on the suppression of DDX3X. Similar to NEAT1 depletion, protein levels of ASC, Caspase-1 and NLRP3 were significantly increased by overexpression of DDX3X. Of note, overexpression of DDX3X exerted the increasing effect on expression of NLRP3 inflammasome-associated proteins which was similar to LPS. However, increased levels of ASC, Caspase-1, NLRP3 and DDX3X were partially ameliorated by Exos/si-Exos in both the LPS and pcDDX3X groups, with the strongest inhibition in the Exos group (Fig. 5B–F, P < *0.05*). The ELISA results showed that similar to the groups treated with LPS, the cytokine concentration of IL-1β was significantly elevated in the groups overexpressed DDX3X compared to control group. In addition, the increasing level of IL-1β in the pc-DDX3X groups was inhibited by Exos and si-Exos, with the Exos group having a more prominent inhibitory activity (Fig. 5G, P < *0.05*).

### HUVECs-derived exosomes inhibited the inflammation by enhancing M2 polarization in vivo

**Fig. 6:**
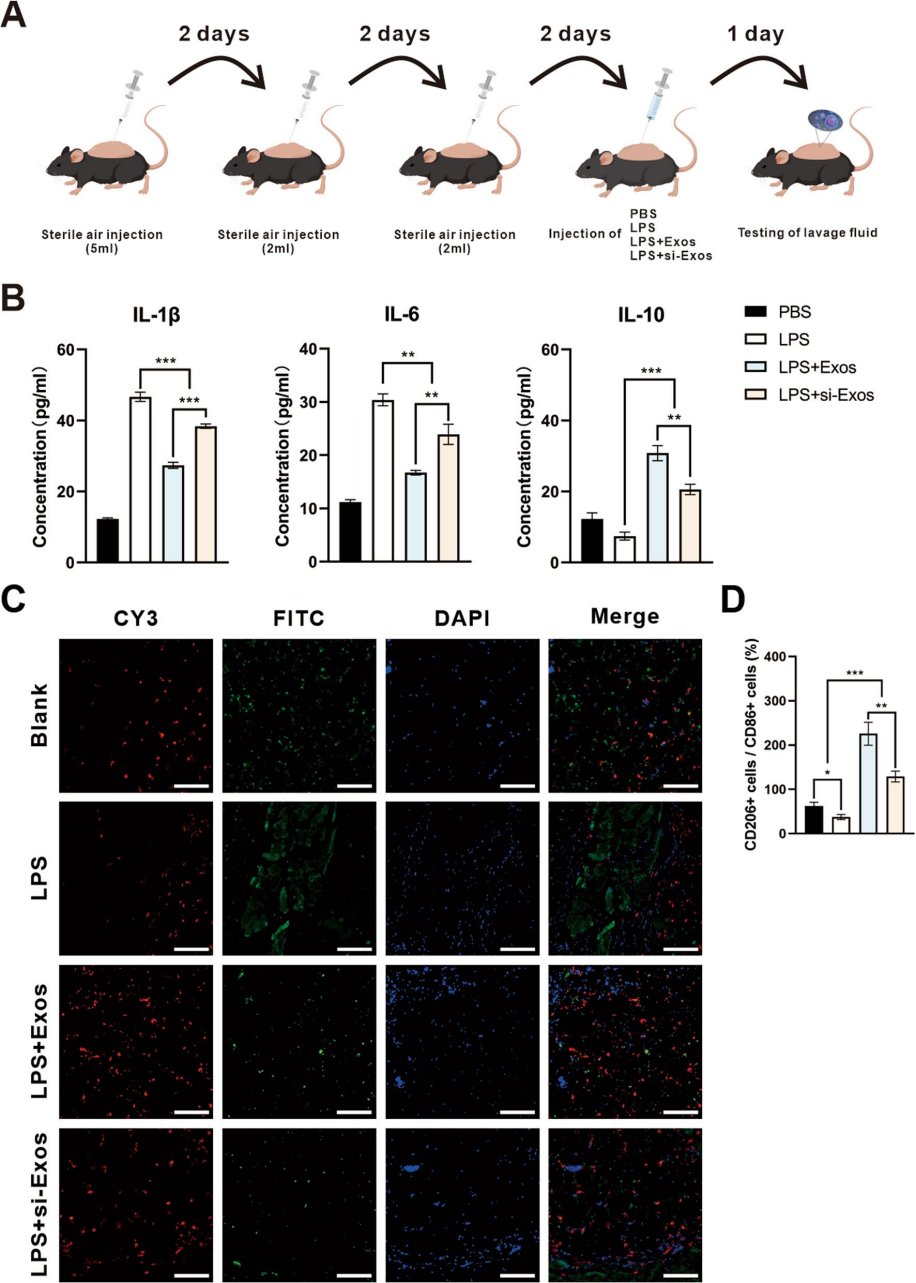
Air pouch model in vivo*.* **A** Schematic illustration of air pouch model establishment. **B** ELISA analysis of IL-1β, IL-6 and IL-10 concentration of lavage fluid. **C** Representative confocal images for CD86 (green) and CD206 (red). The nucleus were counterstained with DAPI (blue). **D** Quantification of the ratio of CD206/CD86 positive cells per field. * P < 0.05, ** P < 0.01, *** P < *0.001*

To assess the effect of the exosomes on polarization of macrophages in vivo, an air pouch model was established (Fig. 6A). The level of inflammation associated cytokines were evaluated by ELISA. IL-1β and IL-6, both for M1 phenotype, were highest, in LPS group, but decreased significantly after the application of Exos and si-Exos. Exos and si-Exos remarkably elevated IL-10 secretion, a marker of M2 polarization, which was significantly reduced subjected to LPS treatment. In particular, the inhibition of NEAT1 reversed the effect of M2 polarization in si-Exos group (Fig. 6B, P < *0. 01*). The results of immunofluorescence staining showed that both M2 macrophages (CD206 labeled, red) and M1 macrophages (CD86 labeled, green) were visible in all groups. The ratio of M2/M1 was significant increased in the group treated with exosomes, especially the exosomes without NEAT1 knockdown (Fig. 6C/D, *P* < *0. 05*).

### Micro-CT and histological analysis of bone regeneration

**Fig. 7:**
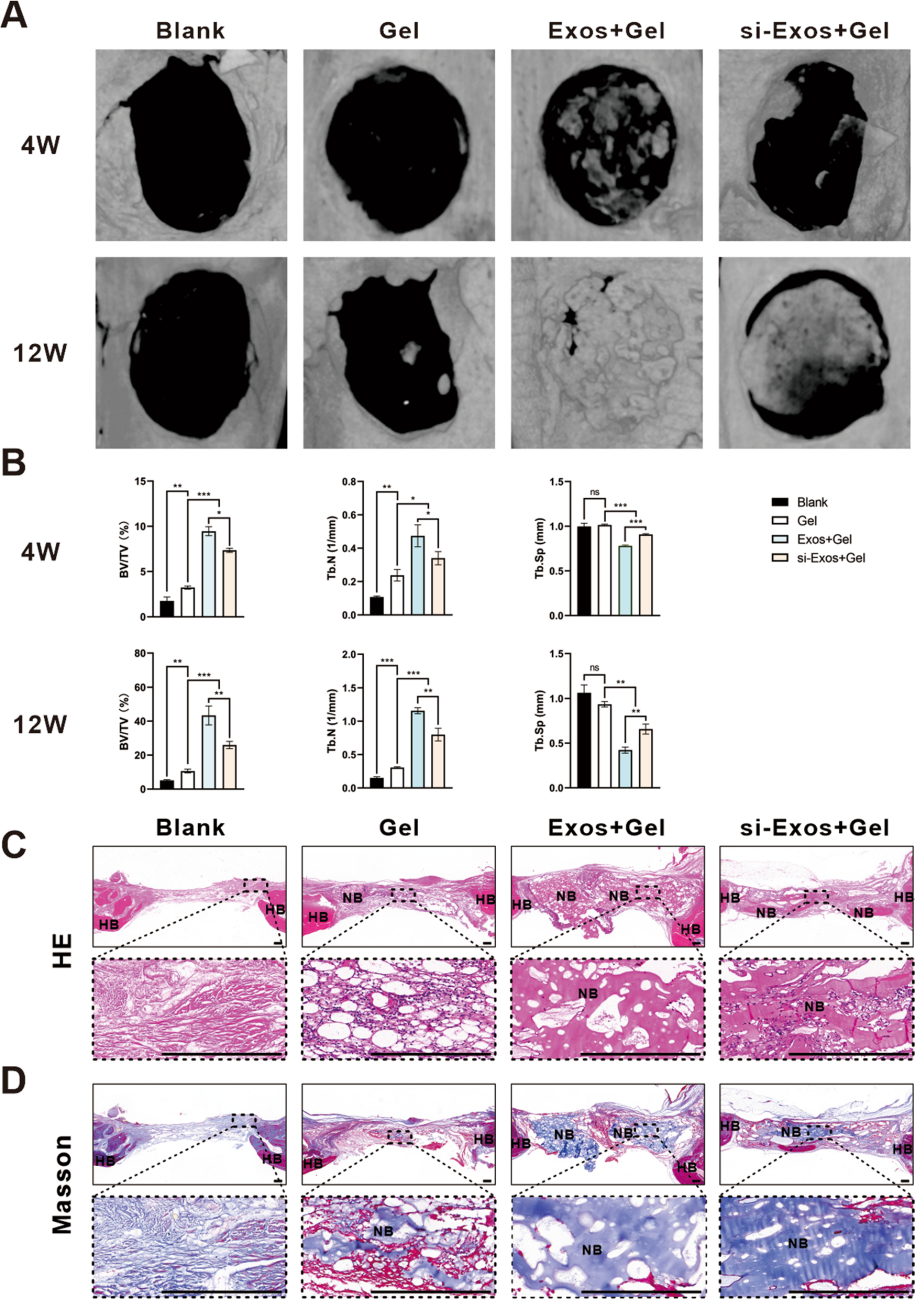
Micro-CT analysis and histological staining of rat bone reconstruction in vivo. **A** 3D reconstruction images of calvarial bone at 4 and 12 weeks postoperatively. **B** Quantitative analysis of BV/TV, Tb. N and Tb. Sp in 3D micro-CT images at 4 and 12 weeks. H&E staining (**C**) and Masson’s trichrome staining (**D**). Scale bar = 200 μm. *HB* host bone; *NB* new bone. * P < 0.05, ** P < 0.01, *** P < *0.001*

The 3D reconstruction of micro-CT images of the rat cranial defects at 4 and 12 weeks were shown in Fig. 7A. The images at 4 weeks showed that sporadic newly formed bone filled the defects of the Exos + Gel and si-Exos + Gel groups, but no obvious bone structure was generated in the control group. With the increase of time after implantation, more new bone was formed. In particular, at 12 weeks, the largest amount of new bone, which nearly filled the calvarial defects, was found in the Exos + Gel group than that the other three groups. Interestingly, little new bone was generated in the control group, while more newly formed bone could be observed in Gel group.

Based on micro-CT images, quantitative analysis including BV/TV, Tb. N and Tb. Sp demonstrated that BV/TV ratio and Tb. N in Exos + Gel and si-Exos + Gel groups were all significantly higher and Tb. Sp was significantly lower than those in the other two groups, with the Exos + Gel group showing a stronger modulation (Fig. 7B, P < *0.05*).

Histological analysis was performed to observe the tissue in the cranial defect area. H&E staining showed an increased deposition of new bone that were generated both along the margin and into the center of calvarial defects after application of the two kinds of exosomes, especially HUVECs-derived exosomes (Fig. 7C). Whereas, in the control group, the most regions of the defects were filled with fibrotic connective tissue with few visible bone regenerations. Masson’s trichrome staining exhibited that in the Gel group, differs from the Blank group, a small amount of new bone can be observable and both Exos + Gel and si-Exos + Gel groups induced a significantly large amount of the osteoid matrix formation and among the four treatments, composite hydrogels with Exos facilitated the most bone regeneration (Fig. 7D).

### Immunohistochemical and immunofluorescence staining of bone regeneration

**Fig. 8:**
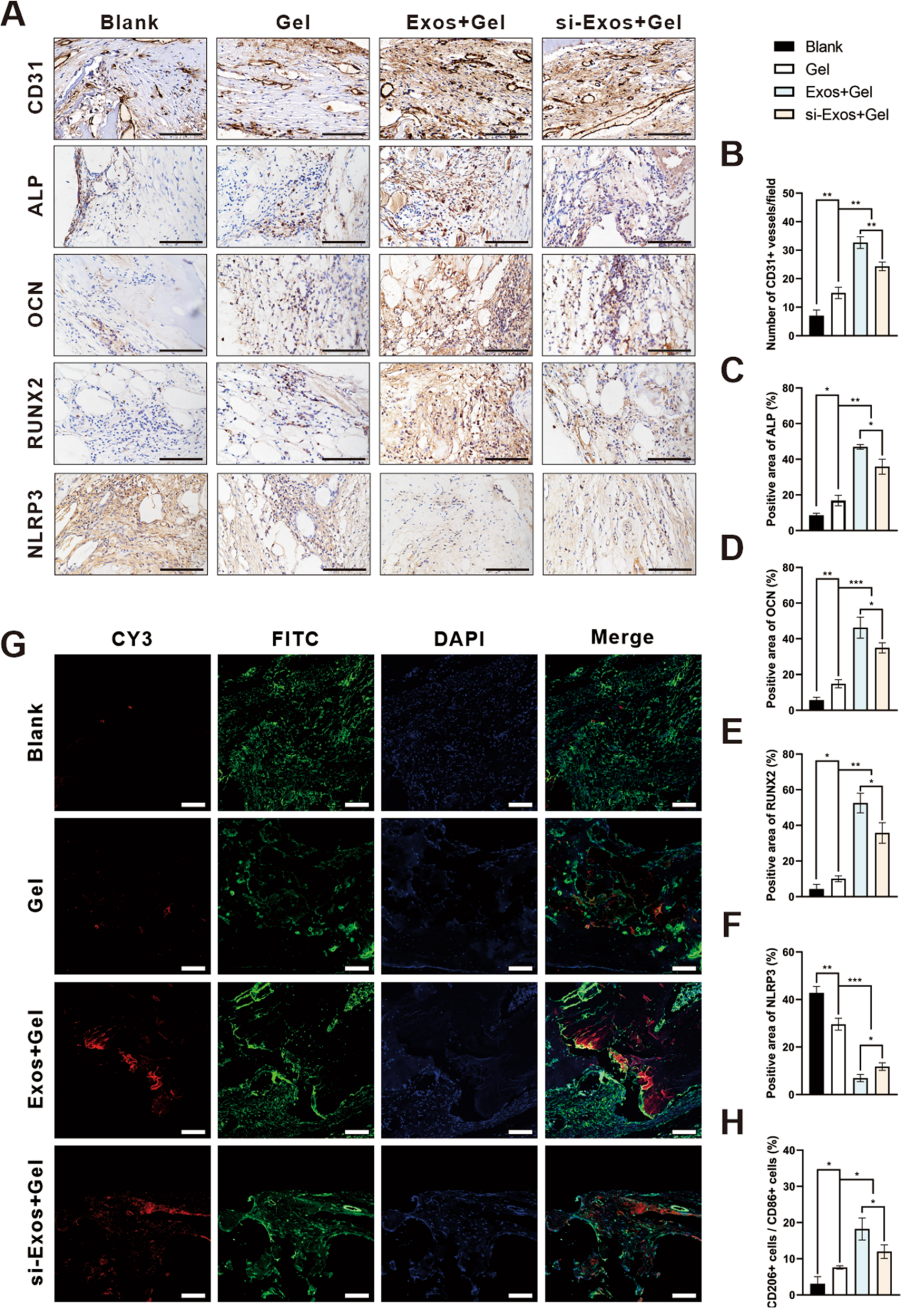
Immunohistochemistry and immunofluorescence staining of bone defect. Representative images of **A** CD31, ALP, OCN, RUNX2 and NLRP3. Quantitative analysis of **B** CD31, **C** ALP, **D** OCN, **E** RUNX2 and **F** NLRP3. **G** Representative confocal images of calvarial sections for CD86 (green) and CD206 (red). In all images the nucleus were counterstained with DAPI (blue). **H** Quantification of the ratio of CD206/CD86 positive cells per field. Scale bar = 100 μm. * P < 0.05, ** P < 0.01, *** P < *0.001*

To further assess the insights into the effect of the exosomes on osteogenesis, immunohistochemical staining of CD31, ALP, OCN, RUNX2 and NLRP3 were performed. The number of CD31-positive vessels was significantly increased in the Exos + Gel group than the other three groups (Fig. 8A/B; *P* < *0.01*). More neovascularization was observed in si-Exos + Gel group compared to Gel and blank groups. Moreover, osteogenic marker ALP, OCN and RUNX2, were more abundantly expressed in both exosomes groups compared to the other two groups, especially in Exos group (Fig. 8A/C–E; *P* < *0.05*). In addition, NLRP3 inflammasome components were significantly decreased in the Exos + Gel and si-Exos + Gel groups, with a much sharper decline in Exos + Gel group, demonstrating that the knockdown of NEAT1 increased the expression of NLRP3 (Fig. 8A/F; *P* < *0.05*).

Moreover, immunofluorescence staining in Fig. 8G revealed, M2 macrophages, labeled by CD206 (red) and M1 macrophages, labeled by CD86 (green), were distributed in the fibrous tissues in all groups. Quantitative analysis indicated that a significant increase of M2/M1 ratio in the Exos + Gel group, indicating a dominant phenotype of M2 polarized macrophages (Fig. 8H; *P* < *0.05*). Although ratio of M2 macrophage was slightly increased in the Gel group, they were still lower than that in the hydrogels composed with exosomes. These effects of Exos were partly compromised by NEAT1 inhibition.

## Discussion

Restoration of bone deficiency is still a clinically challenging problem to treat [29]. Generally, bone regeneration typically undergoes three sequential phases: inflammation, regeneration, and remodeling [30]. The local inflammatory response is crucial for bone tissue regeneration, and an appropriate grade of inflammation can promote bone healing [31, 32]. Recent studies have found that the proportion of M2-polarized macrophages increases during fracture repair, which induce osteogenic differentiation by secreting various growth factors [33, 34]. Among the approaches to modulate M1 to M2 macrophage conversion, strategies including drugs, exosomes, and hydrogels have been exploited to regulate macrophage polarization and promote bone recovery [35, 36]. Exosomes, as emerging cell-free therapies, have received growing attention for their immunomodulatory capabilities [8]. To present, however, very little researches have concerned the effect of exosomes from HUVECs towards osseous restoration, and few studies are available on the mechanisms through which macrophages and specific exosomes stimulate osseous restoration. Therefore, our research sought to unravel the exosomes produced by HUVECs stimulated osteogenesis via involving macrophages through the NEAT1/DDX3X/NLRP3 signaling pathway, through which we found the close association between macrophage polarization and local immune microenviroment, contributing to the bone repair.

Numerous studies in the past have found that exosomes can effectively participate in the immune response to facilitate tissue regeneration [37, 38]. Exosomes from HUVECs were also reported as offering a positive effect in protecting nerve cells from ischemia/reperfusion injury, improving fibroblast photoaging, inducing endothelial progenitor cell homing and inhibiting osteoclast formation to reduce bone resorption [15, 39–41]. Particularly, we demonstrated that HUVEC-derived exosomes could directly promote osteogenic differentiation and increase the migratory capacity of BMSCs, and the aforementioned phenomenon was slightly inhibited with the knockdown of NEAT1 (Additional file 3: Fig. S3). However, the modulatory ability of HUVECs-derived exosomes on immune countermeasure remains largely unknown. In the present study, compared to the conspicuous inflammatory reaction induced by LPS, HUVECs-derived exosomes initiated an obvious alleviated inflammatory reaction characterized by significantly enhanced M2-phenotype polarization of RAW264.7 cells. In accordance, with the advent of polarization, the polarized macrophages further secreted anti-inflammatory cytokines, such as IL-10 and Arg-1. And the level of pro-inflammatory-related cytokines (IL-1β, IL-6) was reduced. Nevertheless, the application of NEAT1 silencer could partially counteracted the trends but not entirely abolish, which further verified the critical role of NEAT1 on M2 polarization. Consistently, Zhang et al*.* found M2 polarization can be promoted by NEAT1 and promotes choroidal neovascularization by sponging miRNA-148a-3p [42]. Besides, NEAT1 accelerates multiple myeloma progression by regulating B7-H3 to promote M2 macrophage polarization [43]. Since there are various bioactivators in exosomes which may also be responsible for M2 phenotype polarization by diverse mechanisms, therefore, NEAT1 inhibitor could only suppress the signaling pathway associated with NEAT1, rendering it incapable of fully eliminating the effect of Exos on promotion of M2 polarization [8, 44, 45].

Emerging evidence has demonstrated that the transition of macrophages to M2 polarization is of great importance on tissue remodeling and able to enhance the osteogenic differentiation and migration of MSCs [46, 47]. It has been widely recognized that M2 polarized macrophages elicit a variety of cytokine including bone morphogenetic protein 2 (BMP-2), IL-10, platelet-derived growth factor (PDGF), transforming growth factor-β (TGF-β) and arginine, which contribute to vascular sprouting and bone healing [48]. In line with these literature studies, our observations suggested that exosomal NEAT1 indirectly enhanced the osteogenic differentiation and migration ability of BMSCs mediated by increased M2 polarized macrophages in a conditioned culture system, which was weakened by NEAT1 silencing. Furthermore, in air pouch model, the involvement of HUVECs-derived exosomes remarkably ameliorated the LPS-induced inflammatory response. With the knockdown of NEAT1, the phenomenon of increase of anti-inflammatory cytokines (IL-10), M2 macrophages (CD206 labeled) and decrease of pro-inflammatory cytokines (IL-6 and IL-1β), M1 macrophages (CD86 labeled) in the lavage fluid was partially reversed. Combining the aforementioned findings*,* we confirmed that exosomal NEAT1 plays an indispensable role in increasing the proportion of M2 macrophages in vitro and in vivo, and further promoted BMSC migration and osteogenic differentiation.

To achieve optimal in vivo application of exosomes in various tissues repair, a suitable carrier to maintain effective local concentration and function of exosomes during the repair process should to be taken into consideration [49, 50]. Hydrogels has generally been presumed a favorable carrier for exosomes because of its similarities to the extracellular matrix (ECM), appropriate physical strength and good biocompatibility [51, 52]. In our release profile, the alginate/GelMA IPN hydrogel represented stable release capacity for HUVECs-derived exosomes with or without NEAT1 inhibition and retained approximately 50% exosomes in the alginate hydrogel during 15 days. Moreover, the composite hydrogels keep releasing exosomes which still maintain the typical cup-like structure at day 15 (Additional file 2: Fig. S2). These results suggest that our synthetic alginate/GelMA IPN hydrogels are reliable vehicles of exosomes to maintain a uniform distribution, sustained release and stable function of exosomes, ultimately achieving the therapeutic goal.

To deeply evaluate the bone repair ability of HUVECs-derived exosomes loaded hydrogels in vivo*,* a rat cranial defect model was employed. Based on the images and quantification of micro-CT analysis, the in vivo application of the exosomes and hydrogel composites markedly enhanced bone regeneration compared to the other groups, especially the naive exosomes without NEAT1 knockdown. Furthermore, the histological and IHC evaluation revealed significant higher levels of angiogenic and osteogenic markers, increased local infiltration of M2 polarized macrophages in the Exos group, leading to a better repair of rat cranial defect, which corresponded to their strongest ability in vitro. Our results are in parallel with the former conclusion that exosomal NEAT1 could promote angiogenesis [15]. Moreover, bone regeneration highly depends on angiogenesis, which is a vital step to restore blood flow providing nutrients, further exerting a positive feedback to bone healing [53]. These encouraging findings supported that exosomal NEAT1 could facilitate bone regeneration in vivo.

In regard to the potential effect of NEAT1 on regulation of macrophages, our study further focused on investigating the downstream molecular mechanism of NEAT1. The noteworthy targets, particularly the NLRP3 inflammasomes, assembled from ASC, caspase-1 and NLRP3, are vital members of the innate immune system [54]. NLRP3 inflammasome plays a significant role in bone inflammation, because of the causal caspase-1 activation and its correlation to inhibition osteogenic adipose accumulation and differentiation in bone tissues [55]. Occupation of NLRP3 inflammasome can accelerate bone resorption, promote osteoclast differentiation and aggravate inflammation, which increase the risk of osteoporosis [56]. However, the regulatory relationship of NEAT1 and NLRP3 still remains to be controversial. Herein we hypothesized that DDX3X worked as a downstream target of NEAT1 to regulate NLRP3 inflammasome activation. Recently, DDX3X has been shown to drive the assembly of NLRP3 inflammasome and determine the fate of cells by interacting with NLRP3 [23]. Knockdown of DDX3X significantly suppressed NLRP3 inflammasome activation caused by LPS and attenuated pyroptosis and cell injury in H9c2 cells [24]. The application of HUVECs-derived exosomes decreased the expressions of ACS, caspase-1, NLRP3 and DDX3X dramatically, which is in consistence with the in vivo findings. Notably, with the use of DDX3X mimic, the level of NLRP3 inflammasome increased highly, and then decreased to some extent when combining the use of the specific exosomes. Thus, these exciting findings supported that HUVECs derived exosomal NEAT1 inhibited NLRP3 inflammasome activation mediated by DDX3X, thereby alleviating LPS-induced inflammation. Coincidentally, it has been suggested that NEAT1 alleviate ischemic stroke inhibiting NLRP3 mediated via miR-10b-5p/BCL6 axis [57]. Likewise, Nong et al*.* indicated NEAT1 could sponge miR-193a-3p via NF-κB signal pathway to alleviate inflammation of normal human fibroblast cells induced by LPS [58]. Conversely, others have demonstrated contrary evidence to those in the present study. The inhibition of NEAT1 could protect endothelial cells from hypoxia-induced NLRP3 inflammasome activation by targeting miR-204/BRCC axis [59]. The reason for these differences is likely to be attributed to the different experimental details and the mechanism network involved, which finally exert diverse effects.

As far as we know, we have taken the first step in the direction that exosomal NEAT1 inhibited LPS-induced inflammatory responses contributing to bone regeneration via the DDX3X/NLRP3 signaling pathway. Regarding to the mutual influence between macrophages and BMSCs, it would be of great importance to gain an in-depth understanding of NEAT1 function involved in the bone healing process. Notably, our study not only proposed a novel cell therapy replacement but also provides a prospective therapeutic strategy to broaden the translational use of HUVECs-derived exosomes for the treatment of clinical bone defects.

## Conclusion

**Fig. 9:**
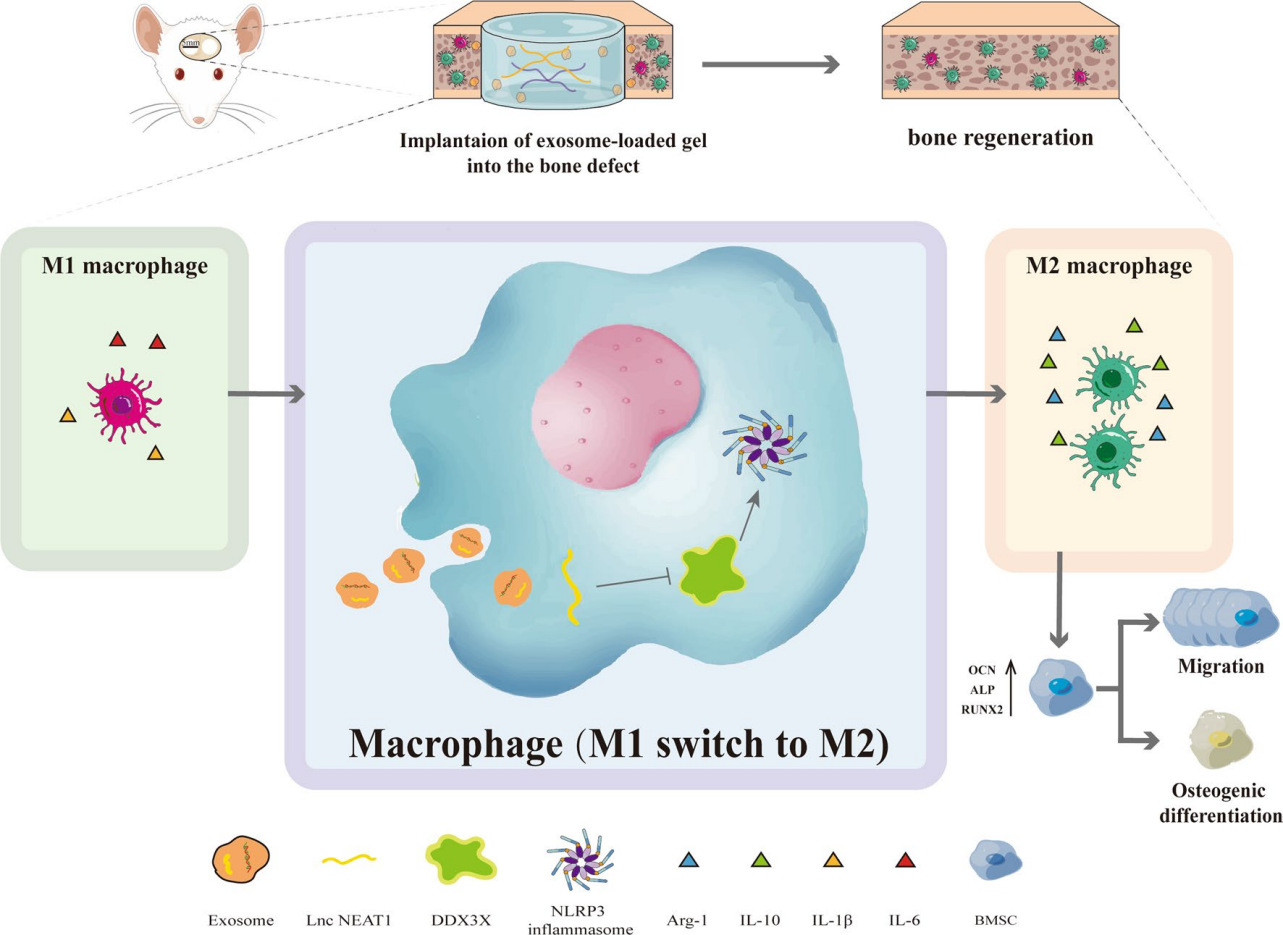
Schematic of the HUVECs derived exosomal NEAT1 mediated bone regeneration mediated by macrophage polarization via DDX3X/NLRP3 axis

Taken together, the role of HUVECs derived exosomal NEAT1 in bone regeneration was unraveled in our current work and the underlying mechanisms were portrayed. Our study clearly indicated NEAT1 can promote M2 polarization and suppress inflammatory responses by modulating the DDX3X/NLRP3 axis, and further promote the osteogenic differentiation and migration potential of BMSC to facilitate bone repair in vivo and in vitro (Fig. 9). The positive impact was suppressed in part by NEAT1 knockdown. Meanwhile, alginate/GelMA IPN hydrogels were verified to provide a reliable delivery platform for exosomes. Therefore, our findings confirmed a promising role of HUVECs derived exosomal NEAT1 as a therapeutic alternative for bone defect.

## Materials and methods

### Cell isolation and culture

HUVECs and murine-derived RAW264.7 cells, macrophage cell line, were both purchased from Cell Bank of Chinese Academy of Sciences (Shanghai, China) and cultured in DMEM medium (HyClone, Logan, UT, USA) and RPMI 1640 medium (HyClone), respectively, containing 1% penicillin–streptomycin solution (Biosharp, Hefei, Anhui, China) and 10% fetal bovine serum (FBS) (Evergreen, Zhejiang, China) at 37 °C with 5% CO_2_.

BMSCs were isolated from neonatal male Sprague–Dawley (SD) rats (5-days-old) according a previous medthod [60]. Briefly, after execution of the rats using the spinal dislocation method, they were then immersed in 75% alcohol for 30 min. The femur and tibia were isolated. Then, the cartilage at both ends of the bone was cut and the bone marrow cavity was repeatedly flushed with culture medium until the bone appeared white. Finally, the rinsed bone marrow tissue was inoculated in a culture dish with 6 mL of culture medium (L-DMEM containing 10% FBS, 1% penicillin and streptomycin). The culture was incubated at 37 °C and the culture medium was changed every 3 days. The passages of BMSC used in studies were 1 to 3.

### Transfection assay

LncRNA NEAT1 expression in HUVECs was knocked down using NEAT1 siRNA lentivirus, synthesized by Wuhan Biofavor Biothech Service Co., with Lipofectamine 3000 Transfection Reagent (Invitrogen, Carlsbad, CA, USA) according to the manufacturer’s instructions. The sequences of NEAT1 siRNA were as follow: forward: GCC TTG TAG ATG GAG CTT GC; reverse: GCA CAA CAC AAT GAC ACC CT. RAW264.7 were inoculated on 6-well plates and cultured overnight. The sequence of DDX3X (Additional file 4: Table S1) was provided by Huayan Biotechnology Co., LTD (Wuhan, China) and the plasmids pcDNA3.1-DDX3X were constructed. The plasmids were introduced into RAW264.7 using Lipofectamine 3000 Transfection Reagent (Invitrogen) according to the manufacturer's instructions. Transfection efficiency was verified by qRT-PCR, and cells were collected for subsequent experiments.

### Extraction and identification of exosomes

Exosomes, including Exos and si-Exos, were both isolated. Specifically, HUVECs were pretreated by transfecting with NEAT1 siRNA (si-NEAT1-HUVECs). Afterwards, the mediums were changed to exosome free mediums and cultured for 72 h when the confluence reached 80–90%. The supernatant of HUVECs and si-NEAT1-HUVECs group was collected for extracting exosomes. Cells and cell debris were first removed by centrifugation at 300 × g for 10 min and then at 2,000 × g for 10 min. Then, centrifugation at 10,000 × g for 30 min and 0.22 μm sterilized filters (MilliporeExpress® PES membrane, Millex, Bedford, MA, USA) were used to remove the larger diameter extracellular vesicles. Finally, the exosomes were obtained by centrifugation at 110,000 × g for 70 min and resuspended with pre-cooled PBS. The exosomes were lysed with radio-immunoprecipitation assay (RIPA) lysis buffer, and the protein concentration in the exosomes was detected with the bicinchoninic acid (BCA) protein assay kit (Servicebio, Wuhan, Hubei, China) according to the manufacturer's instructions. qRT-PCR assay was applied to detect the expression level of NEAT1 in Exos and si-Exos.

The exosomes were identified by the marker proteins CD81, CD63, and TSG101 by western blotting. Morphological identification was observed by Transmission electron microscopy microscope (TEM, Tecnai G2 20, Thermo Fisher Scientific, Cleveland, OH, USA). The particle size distribution and nanoparticle concentration were evaluated by NTA using ZetaView (Particle Metri, Bavaria, Germany). Three 60 s video recordings of the Brownian motion of the exosomes were taken while the blue laser (488 nm) irradiated the exosomes, and finally the NTA software analysis was performed using ZetaView software (version 8.02.31, Particle Metri, Bavaria, Germany).

### Internalization of exosomes

To trace the internalization of exsomes in macrophages, exosomes were labeled with Dil dye (Solarbio, Beijing, China). Then, the Dil-labeled exosomes were co-cultured with RAW264.7 cells for 3 h. The cells were stained with phalloidin-FITC (C1033, Beyotime Biotechnology, Shanghai, China) and DAPI (C1002, Beyotime Biotechnology) to visualize the cytoskeleton and nucleus, respectively. The uptake was photographed with a confocal microscope (Nikon, Tokyo, Japan).

### Effects of conditioned media on BMSC responses

A conditioned culture system was designed to investigate the effects of exosomes-induced macrophages on the osteogenic differentiation of BMSCs. To obtain the CM from macrophages, RAW264.7 macrophages were seeded in 6-well plate at a density of 2 × 10^5^/well and achieved a confluence of 70–80%. Then, the cells were cultured in normal complete medium containing 100 ng/mL LPS (Biosharp) for 24 h. Thereafter, Exos and si-Exos (100 μg/mL) were added to study the effect of exosomes on the polarization of macrophages, respectively. At day 3, the supernatant of the macrophages in four groups (PBS, LPS (100 ng/mL), LPS + Exos (100 μg/mL) and LPS + si-Exos (100 μg/mL)) was collected, centrifuged at 1200 rpm for 10 min to remove precipitates, then mixed with osteogenic medium (HyCyte, Jiangsu, Suzhou, China) at a ratio of 1:1 (v/v) to obtain the mixed RAW264.7 CM. Specifically, BMSCs were seeded at a density of 2 × 10^5^ cells/well in 6-well plate. After 24 h, the culture medium of BMSCs was replaced with different RAW264.7 CM. At day 7 and 14, the total RNA of BMSCs in conditioned culture system were extracted. The expression of osteogenic related genes and protein was detected by qRT-PCR and western blotting. When the culture reaches day 7, ALP staining was performed using the BCIP/NBT alkaline phosphatase color development kit (C3206, Beyotime Biotechnology) according to the instructions provided by the manufacturer.

### Transwell migration assay

BMSCs were seeded in the upper chamber at a density of 5 × 10^4^ in a transwell plate (24-well plate, JETBIOFIL, Guangzhou, Guangdong, China). 600 μL conditioned medium derived from the macrophages (PBS; LPS (100 ng/mL); LPS + Exos (100 μg/mL) and LPS + si-Exos (100 μg/mL)) were added to the lower chamber. After incubation for 24 h, the cells remained on the upper side of the chamber were removed. The cells which migrated to the lower chamber were stained with DAPI (Beyotime Biotechnology). Images were observed by a confocal microscope (Nikon) and analyzed with ImageJ software (National Institutes of Health, USA). The three fields of view per well were analyzed by counting the number of DAPI-labeled positive staining cells.

### Preparation of alginate/GelMA IPN Hydrogel and exosome-hydrogel composite

To prepare the alginate/GelMA IPN hydrogel, 200 mg alginic acid sodium salt (SA) was dissolved in 10 mL calcium-free dulbecco phosphate-buffered saline (DPBS) solution (Procell, Wuhan, Hubei, China) to obtain SA solution. 500 μL SA solution was mixed with photo-initiator lithium phenyl-2,4,6-trimethylbenzoylphosphinate (LAP, StemEasy, Jiangsu, China) to the target concentration of 0.5% (m/v) LAP and 1% (m/v) SA. Afterwards, 100 mg gelatin methacryloyl (GelMa) was added into the mixture and then put in 70 °C for 30 min until completely dissolved to obtain the hybrid 1% (m/v) alginate/10% (m/v) GelMA IPN-hybrid hydrogel. 100 μg exosomes (including Exos and si-Exos) were resuspended in 100 μL hybrid hydrogel in the dark at 37℃ to produce the exosomes + Gel mixed solutions. Then, the mixed solutions were photopolymerized after exposed to ultraviolet (UV) light for 30 s and subsequently immersed in 2% (m/v) CaCl_2_ solution for crosslinking to finally obtain exosomes + Gel composites. In order to measure the distribution of exosomes in hydrogel, exosomes were labeled with Dil and then observed by the confocal microscope (Nikon).

### The exosome retaining ability of hydrogels

The release profile of exosomes from alginate/GelMA IPN hydrogel was evaluated using BCA protein assay kit [61] and TEM. Briefly, 100 μL of the above prepared exosome/ hydrogels composites containing 100 μg exosomes were placed in a 96-well plate supplemented with 100 μL PBS. Subsequently, the supernatant in the well was collected and the well was refilled with another 100 μL PBS at specific time points. The protein concentration in PBS collected was measured to draw a release curve. Morphological characterization of exosomes released from the hydrogel composites was observed by TEM.

### Flow cytometry

In order to identify the polarization of RAW264.7, LPS-preconditioned cells were incubated in medium containing Exos or si-Exos (100 μg/mL). Subsequently, treated cells with TruStain Fc X™ PLUS (156603, Biolegend, USA) and Triton X-100 (P0096-100 ml, Beyotime Biotechnology), then they were incubated with the antibodies. Thereafter, the cells were analyzed by flow cytometry (BD LSR FortessaTM X-20, San Jose, USA) utilizing the flowJo software (version 10.7.1, Stanford University, USA). Flow cytometry analysis was performed on each sample in triplicate. The antibodies were used as follows: Phycoerythrin (PE) conjugated-CD86 (12-0862-82, eBioscience, USA), PE/Cyanine7 conjugated-CD206 (141719, Biolegend).

### Quantitative real-time polymerase chain reaction

TRIzol reagent (Invitrogen) was utilized to extract total RNA. cDNA was synthesized by RevertAid First Strand cDNA Synthesis Kit (K1622, Thermo Fisher Scientific) following the manufacturer’s instructions. The levels of mRNA were calculated and normalized to GAPDH using the 2 ^−ΔΔCt^ method. The primer sequences were listed in Additional file 5: Table S2.

### Western blot analysis

Total protein was measured by the BCA Protein Detection Kit (Thermo Fisher Scientific). The proteins were extracted by gradient sodium dodecyl sulfate polyacrylamide gel electrophoresis (SDS-PAGE) and then transferred to nitrocellulose membranes. After being blocked by 5% skimmed milk, the membrane was incubated at 4 °C along with the primary antibodies overnight, followed by incubation with horseradish peroxidase (HRP)-conjugated secondary antibodies at room temperature for 1 h. Band intensities was analyzed using Image-Pro Plus (Media Cybernetics, USA) by densitometry. β-actin was used as an internal control. The following primary antibodies were used: NLRP3 (1:1000, ab214185; Abcam, Cambridge, UK), IL-1β (1:1000, A16288, ABclonal, Woburn, MA, USA), Caspase-1 (1:1000, ab207802, Abcam), the adaptor protein apoptosis-associated speck-like protein containing a CARD (ASC; 1:1000, ab180799, Abcam), DDX3X (1:1000, ab235940, Abcam), GAPDH (1:5000, ab8245, Abcam) and β-actin (1:5000, ab8227, Abcam).

### Enzyme-linked immunosorbent assay

Cell supernatants of RAW264.7 cells incubated on 24-well plates were treated with PBS, LPS, LPS + Exos and LPS + si-Exos for 24 h and 4 groups (PBS, LPS (100 ng/mL), LPS + Exos (100 μg/mL) and LPS + si-Exos (100 μg/mL)) lavage fluid from air pouch were collected for detecting IL-6, IL-1β and IL-10 by ELISA kits (Sigma-Aldrich, St. Louis, MO, USA), respectively, according to the manufacturer’s specifications. Besides, the supernatants of RAW264.7 cells treated with PBS, LPS, LPS + Exos, LPS + si-Exos, pc-DDX3X, pc-DDX3X + Exos, and pc-DDX3X + si-Exos were collected respectively for detection of IL-1β by ELISA kits (Sigma-Aldrich).

### Animal experiments

In our study, All the procedures were approved by the guidelines of the Animal Research Committee of the Huazhong University of Science and Technology.

### Air pouch assay in vivo

12 C57BL/6 mice (40 g; 10 weeks old; male) were used and randomly assigned to four groups (n = 4): PBS, LPS, LPS + Exos and LPS + si-Exos. The mouse were anesthetized by intraperitoneal injection of 5% pentobarbital sodium (Sigma-Aldrich) at 0.5 mL/kg before surgery, and injectied 5 mL sterilized air into loose dorsal tissue and supplemented with 2 mL of sterile air on days 3 and 5 to establish a stable air pouch model (Fig. 0.6A). At day 7, 2 mL PBS, LPS (100 ng/mL), LPS + Exos and LPS + si-Exos (100 μg/mL) were injected subcutaneously. After 24 h, 2 mL PBS was applied for washing the subcutaneous pouch to collect the lavage fluid. The levels of IL-6, IL-10 and IL-1β were detected by ELISA. Afterwards, the mice were sacrificed. All airpouches were harvested and processed for immunofluorescence staing to determine the inflammatory response by the number of infiltrated macrophages.

### Cranial defect rat model establishment in vivo

16 Sprague–Dawley rats (400 g; 8 weeks old; male) were used for this operation and randomly assigned to four groups (n = 4): (1) alginate/GelMA IPN hydrogel groups (Gel): 40 μL hydrogel; (2) HUVECs-Exos + hydrogel (Exos + Gel): 40 μg HUVECs-Exos dissolved in 40 μL hydrogel; (3) si-NEAT1-Exos/hydrogel (si-Exos + Gel): 40 μg si-Exos dissolved in 40 μL hydrogel; (4) control group: 40 μL PBS. All rats were anaesthetized by an intraperitoneal injection of 5% pentobarbital sodium (Sigma–Aldrich) at a dose of 0.5 mL/kg prior to surgery. Afterwards, two full-thickness critical size of cranial defects (5 mm in diameter) were created symmetrically on each side of the rat’s cranium with a trephine bur. The different components of implant materials (5 mm in diamter and 1 mm in depth) were prepared in advance and gently implanted into the cranial defect according to the grouping, respectively. Afterwards, the soft tissues were closed and the skin was sutured. Animals were euthanized at 4 and 12 weeks postoperatively to harvest specimens from the defect sites for the further study.

### Micro-computed tomography (micro-CT)

The cranium defect sites were evaluated via Micro-CT (SkyScan 1176). The 3D bone reconstruction images were obtained by using CTvox software (version 3.1.1, Bruker). As the density of bone tissue and hydrogel differs, the MicroView software (Bruker version 1.15.4) was used to distinguish one from the other. The percentage of new bone volume/total tissue volume (BV/TV), bone trabecular separation (Tb. Sp) and bone trabecular number (Tb. N) were analyzed.

### Histology, immunohistochemistry and immunofluorescence analysis

The harvested specimens were fixed, decalcified, gradually dehydrated, and embedded in paraffin. Samples were sectioned into 3 μm. Haematoxylin and Eosin (H&E) and Masson’s trichrome staining was performed for the observation of osteogenesis in calvarial bone defects. For immunohistochemistry staining (IHC), the sections were incubated with anti-rat CD31 antibody (1:1000, 28083-1-AP, PTG, Chicago, USA) to label neovascularization and anti-rat antibodies against ALP (1:100, 11400-1-AP, PTG), OCN (1:200, 23418-1-AP, PTG) and RUNX2 (1:1000, 20700-1-AP, PTG) to characterize the new bone in the tissue, followed with 3,3-diaminobenzidine substrate (DAB, Vector Laboratories, Burlingame, CA 4 min), and counterstained with Mayer’s hematoxylin (Sigma-Aldrich). Slides were imaged using a Leica microscope (Leica, Wetzlar, Germany). ImageJ software (National Institutes of Health) was used to analyze CD31 positive vessels and calculated the area of ALP, OCN and RUNX2-positive region in each field of view. In each section of each sample, at least three sections and three areas were randomly selected for observation and analysis.

For immunofluorescence staining, to detect the macrophages, the sections were incubated with primary antibodies against CD86 (1:100, A2353, Abclonal) and CD206 (1:2000, 60143-1-IG, PTG) overnight at 4 °C, followed with secondary antibodies (Goat Anti-Rabbit IgG H&L (HRP), ab205718, Abcam, Goat Anti-Mouse IgG H&L (HRP), ab6789, Abcam) at room temperature for 1 h in the dark. Cell nucleus were stained with DAPI (Beyotime Biotechnology) for 20 min. Images were acquired by a confocal microscope (Nikon). The ratio of CD206-positive cells to CD86-positive cells was analyzed using ImageJ software (National Institutes of Health).

### Statistical analysis

All Results are expressed as mean ± SD derived from at least three independent experiments. One way analysis of variance (ANOVA) and unpaired t-test were used to assess the differences between groups with GraphPad Prism 8.0 software (San Diego, CA, USA). Statistical significance was shown as follows: * *p* < 0.05, ** *p* < 0.01, *** *p* < 0.001, **** *p* < 0.0001; ns, no statistically significant difference (p > 0.05).

## Supplementary Information

**Additional file 1: Figure S1.** Quantification of NEAT1 level in Exos or si-Exos. *** P < 0.001.

**Additional file 2: Figure S2.** Observation of the morphology of Exos and si-Exos released from the hydrogel at day 15 under TEM. Scale bar = 200nm.

**Additional file 3: Figure S3.** Exos/si-Exos promoted osteogenic differentiation and migration of BMSCs. (A) qRT-PCR analysis for mRNA expressions of ALP, OCN and RUNX2 on day 7. (B) Western blot analysis and quantification. (C) of protein levels of ALP, OCN and RUNX2 on day 7. (D) Representative images of transwell assay and quantification (F) of cell migration. Images (E) and quantification (G) of ALP staining after 7 days of osteogenic induction. Scale bar = 200 μm. * P < 0.05, ** P < 0.01, *** P < 0.001.

**Additional file 4: Table S1.** Construction of Lentiviral Overexpression Vector.

**Additional file 5: Table S2.** Primer sequences employed for reverse transcription-quantitative polymerase chain reaction.

## Data Availability

The datasets used and/or analyzed during the current study are available from the corresponding author on reasonable request.

## Abbreviations

DDX3X: DEAD-Box Helicase 3 X-Linked
NLRP3: Nod-like receptor protein 3
NLRC4: NLR family CARD domain containing 4
TNF-α: Tumor necrosis factor-α
lncRNAs: Long non-coding RNAs
NEAT1: Nuclear enrichment enriched transcript 1
IL-6: Interleukin-6
IL-1β: Interleukin-1β
IL-10: Interleukin-10
TGF-β: Transforming growth factor-β
HUVECs: Human vascular endothelial cells
IPN: Interpenetrating polymer network
LPS: Lipopolysaccharide
GelMA: Gelatin methacrylate
MSCs: Mesenchymal stem cells
BMSCs: Bone marrow-derived mesenchymal stem cells
Exos: Exosomes derived from HUVECs
si-Exos: Exosomes derived from si-NEAT1-HUVECs
RIPA: Radio-immunoprecipitation assay
BCA: Bicinchoninic acid
TEM: Transmission electron microscope
NTA: Nanoparticle tracking analysis
CM: Conditioned medium
CCK8: Cell counting kit-8
UV: Ultraviolet
ASC: Associated speck-like protein containing a caspase recruitment domain
GAPDH: Glyceraldehyde-3-phosphate dehydrogenase
ELISA: Enzyme-linked immunosorbent assay
micro-CT: Micro-Computed Tomography
BV/TV: New bone volume/total tissue volume
Tb. Sp: Bone trabecular separation
Tb. N: Bone trabecular number
H&E: Hematoxylin and eosin
IHC: Immunohistochemistry staining
RUNX2: Runt-related transcription factor 2
OCN: Osteocalcin
ALP: Alkaline phosphatase
qRT-PCR: Quantitative real-time polymerase chain reaction
miRNAs: MicroRNAs
FITC: Fluorescein isothiocyanate
PE: Phycoerythrin
Cy7: Cyanine 7
DAPI: 4’,6-Diamidino-2-phenylindole
