# Supplementary figures and images for: Exosomal Lnc NEAT1 from endothelial cells promote bone regeneration by regulating macrophage polarization via DDX3X/NLRP3 axis
Source: J Nanobiotechnology. 2023 Mar 20;21:98. doi: 10.1186/s12951-023-01855-w (PMC10029245; doi:10.1186/s12951-023-01855-w)

**Figure S1：**

**
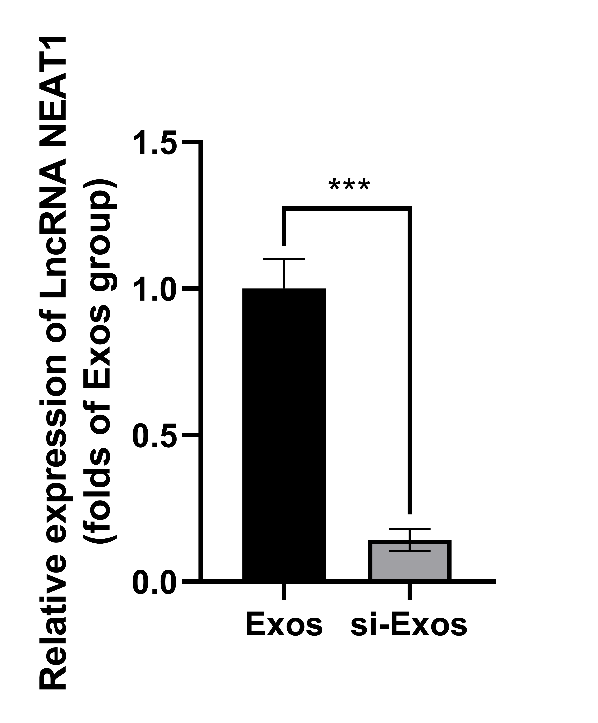
**

Figure S1: Quantification of NEAT1 level in Exos or si-Exos. *** P < 0.001

Supplement: Supplementary file 1 — Additional file 1: Figure S1. Quantification of NEAT1 level in Exos or si-Exos. *** P < 0.001. [file 12951_2023_1855_MOESM1_ESM.docx]
