# Supplementary material for: Exosomal Lnc NEAT1 from endothelial cells promote bone regeneration by regulating macrophage polarization via DDX3X/NLRP3 axis
Source: J Nanobiotechnology. 2023 Mar 20;21:98. doi: 10.1186/s12951-023-01855-w (PMC10029245; doi:10.1186/s12951-023-01855-w)
Supplement: Supplementary file 2 — Additional file 2: Figure S2. Observation of the morphology of Exos and si-Exos released from the hydrogel at day 15 under TEM. Scale bar = 200nm. [file 12951_2023_1855_MOESM2_ESM.docx]

**Figure S2：**

**
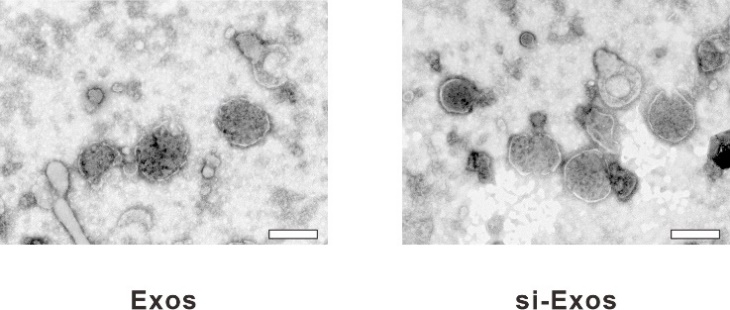
**

Figure S2: Observation of the morphology of Exos and si-Exos released from the hydrogel at day 15 under TEM. Scale bar = 200nm
