# Supplementary material for: Exosomal Lnc NEAT1 from endothelial cells promote bone regeneration by regulating macrophage polarization via DDX3X/NLRP3 axis
Source: J Nanobiotechnology. 2023 Mar 20;21:98. doi: 10.1186/s12951-023-01855-w (PMC10029245; doi:10.1186/s12951-023-01855-w)
Supplement: Supplementary file 3 — Additional file 3: Figure S3. Exos/si-Exos promoted osteogenic differentiation and migration of BMSCs. (A) qRT-PCR analysis for mRNA expressions of ALP, OCN and RUNX2 on day 7. (B) Western blot analysis and quantification. (C) of protein levels of ALP, OCN and RUNX2 on day 7. (D) Representative images of transwell assay and quantification (F) of cell migration. Images (E) and quantification (G) of ALP staining after 7 days of osteogenic induction. Scale bar = 200 μm. * P < 0.05, ** P < 0.01, *** P < 0.001. [file 12951_2023_1855_MOESM3_ESM.docx]

**Figure S3：**


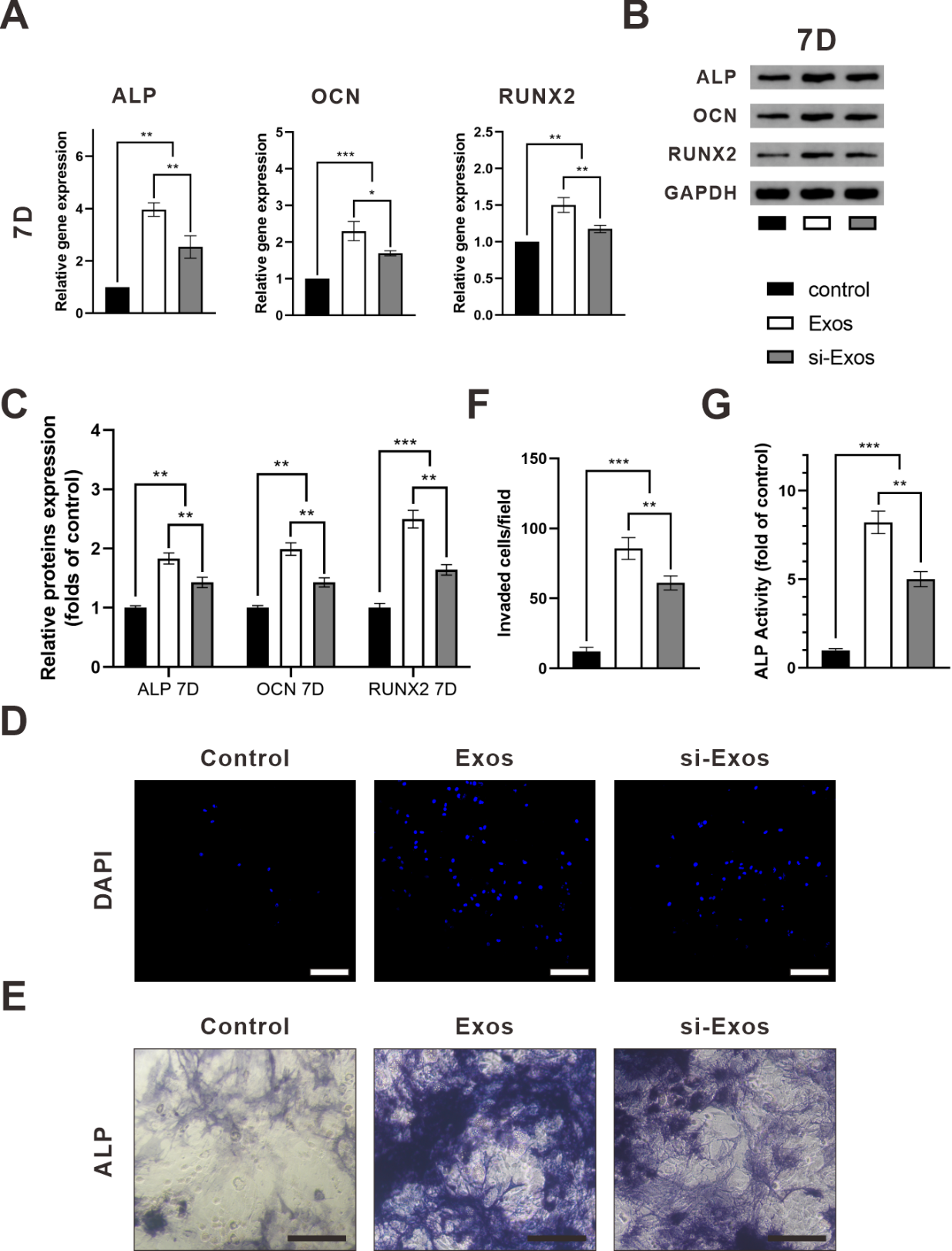


Figure S3: Exos/si-Exos promoted osteogenic differentiation and migration of BMSCs. (A) qRT-PCR analysis for mRNA expressions of ALP, OCN and RUNX2 on day 7. (B) Western blot analysis and quantification. (C) of protein levels of ALP, OCN and RUNX2 on day 7. (D) Representative images of transwell assay and quantification (F) of cell migration. Images (E) and quantification (G) of ALP staining after 7 days of osteogenic induction. Scale bar = 200 μm. *** *P < 0.05, ** P < 0.01, *** P < 0.001.*
