# Supplementary material for: Exosomal Lnc NEAT1 from endothelial cells promote bone regeneration by regulating macrophage polarization via DDX3X/NLRP3 axis
Source: J Nanobiotechnology. 2023 Mar 20;21:98. doi: 10.1186/s12951-023-01855-w (PMC10029245; doi:10.1186/s12951-023-01855-w)
Supplement: Supplementary file 4 — Additional file 4: Table S1. Construction of Lentiviral Overexpression Vector. [file 12951_2023_1855_MOESM4_ESM.docx]

**Table S1.** Construction of Lentiviral Overexpression Vector

| Gene | DDX3X |
| --- | --- |
| Method | The DDX3X gene sequence of mouse origin was synthesized, and EcoRI-XbaI restriction sites (Red) were added at both ends. The resulting gene fragment was blunt-ended cloned into the PUC57 vector. |
| DDX3X  sequence | GGATCCatgagtcatgtggcagtggaaaatgcgctcgggctggaccagcagtttgctggcctagacctgaactcttcagataatcagagtggaggaagtacagcaagcaaagggcgttatatcccacctcatttaaggaacagagaagctactaaaggattctatgacaaagacagttcagggtggagttctagtaaagataaggatgcatacagcagttttggatcacggggtgattcaagagggaagtctagcttctttggagatcgtggaagtggatcaaggggaaggtttgatgatcgtggacggggagactatgatggcattggtggccgtggagatagaagtggctttggcaaatttgaaagaggtggaaatagtcgctggtgtgacaaatcagatgaagatgactggtcaaagccactcccaccaagtgaacgattggaacaggaactcttttctggaggcaatactgggattaactttgagaaatatgatgacattccagtcgaagcaacaggcaacaactgtcctccacacattgaaagtttcagtgatgtcgagatgggagaaattattatgggaaacattgagcttactcgttatactcgcccaactccagtgcagaagcatgctattcctattatcaaagagaaaagagacttgatggcttgtgctcaaacaggctctggaaaaactgcagcatttctcttgcccatcttgagtcagatctatgctgatggtccaggagaagctctgagggctatgaaggaaaatggaagatatggccgtcgtaaacagtatccaatctctttggtactggcaccaacgagagaattggcagtgcagatctatgaggaagccagaaaattctcataccgatctagagtccgtccttgcgtggtttatggtggtgctgaaattggccagcagattcgagacttagaacgtggatgccacttgttagtagccactccaggacgtctagtggatatgatggagagagggaagatcgggttagacttctgcaaatacctggtgttagatgaagctgaccggatgttagatatggggtttgaacctcagatacgaagaatagttgaacaagacactatgcctccaaaaggtgtccgccacactatgatgtttagtgctacttttcctaaggaaatacagatgctggcccgtgatttcttagatgagtacatatttctggctgtaggaagagttgggtctacttcagagaacatcacacaaaaagtggtttgggtggaggagatagacaaaaggtcatttctgcttgaccttctaaatgcaacaggcaaggattccctgactctagtgtttgtggagaccaaaaagggggcagattcgctggaggatttcttataccatgaaggatatgcttgtaccagtatccatggagaccgttctcagagagatagggaagaggcccttcaccagttccgctcaggaaaaagcccaattctagtggctacagcagtagcagcaagaggactggatatttcaaatgtgaagcatgttattaattttgacctgcctagtgatatcgaagaatatgtgcatcgcataggccgtacaggccgtgtgggaaaccttggtcttgccacctcattctttaatgaaaggaatataaatatcacaaaggatttactggatcttcttgttgaagcaaaacaagaagtgccttcttggttagagaacatggcttttgaacaccactacaagggtagcagtcgtggacgttctaagagcagtcgatttagtggagggtttggtgccagagactaccgacagagtagcggtgccagcagttccagcttcagcagcagccgtgcaagcagcagtcgaagtggtggaggtggccatggcggcagtcgaggatttggtggaggtggctacggaggcttttacaacagtgatggatatggagggaattataactcccagggggttgactggtggggtaacTCTAGA |
|  | |
| pcDNA3.1-mDDX3X plasmid | |
|  | mDDX3X-Myc  *Tag sequence was Yellow highlighted and gene sequence was green highlighted. |
| pcDNA3.1-mDDX3X plasmid sequence | CATGACCTTATGGGACTTTCCTACTTGGCAGTACATCTACGTATTAGTCATCGCTATTACCATGGTGATGCGGTTTTGGCAGTACATCAATGGGCGTGGATAGCGGTTTGACTCACGGGGATTTCCAAGTCTCCACCCCATTGACGTCAATGGGAGTTTGTTTTGGCACCAAAATCAACGGGACTTTCCAAAATGTCGTAACAACTCCGCCCCATTGACGCAAATGGGCGGTAGGCGTGTACGGTGGGAGGTCTATATAAGCAGAGCTCTCTGGCTAACTAGAGAACCCACTGCTTACTGGCTTATCGAAATTAATACGACTCACTATAGGGAGACCCAAGCTGGCTAGTTAAGCTTGGTACCGAGCTCGGATCCGCCACCATGAGTCATGTGGCAGTGGAAAATGCGCTCGGGCTGGACCAGCAGTTTGCTGGCCTAGACCTGAACTCTTCAGATAATCAGAGTGGAGGAAGTACAGCAAGCAAAGGGCGTTATATCCCACCTCATTTAAGGAACAGAGAAGCTACTAAAGGATTCTATGACAAAGACAGTTCAGGGTGGAGTTCTAGTAAAGATAAGGATGCATACAGCAGTTTTGGATCACGGGGTGATTCAAGAGGGAAGTCTAGCTTCTTTGGAGATCGTGGAAGTGGATCAAGGGGAAGGTTTGATGATCGTGGACGGGGAGACTATGATGGCATTGGTGGCCGTGGAGATAGAAGTGGCTTTGGCAAATTTGAAAGAGGTGGAAATAGTCGCTGGTGTGACAAATCAGATGAAGATGACTGGTCAAAGCCACTCCCACCAAGTGAACGATTGGAACAGGAACTCTTTTCTGGAGGCAATACTGGGATTAACTTTGAGAAATATGATGACATTCCAGTCGAAGCAACAGGCAACAACTGTCCTCCACACATTGAAAGTTTCAGTGATGTCGAGATGGGAGAAATTATTATGGGAAACATTGAGCTTACTCGTTATACTCGCCCAACTCCAGTGCAGAAGCATGCTATTCCTATTATCAAAGAGAAAAGAGACTTGATGGCTTGTGCTCAAACAGGCTCTGGAAAAACTGCAGCATTTCTCTTGCCCATCTTGAGTCAGATCTATGCTGATGGTCCAGGAGAAGCTCTGAGGGCTATGAAGGAAAATGGAAGATATGGCCGTCGTAAACAGTATCCAATCTCTTTGGTACTGGCACCAACGAGAGAATTGGCAGTGCAGATCTATGAGGAAGCCAGAAAATTCTCATACCGATCTAGAGTCCGTCCTTGCGTGGTTTATGGTGGTGCTGAAATTGGCCAGCAGATTCGAGACTTAGAACGTGGATGCCACTTGTTAGTAGCCACTCCAGGACGTCTAGTGGATATGATGGAGAGAGGGAAGATCGGGTTAGACTTCTGCAAATACCTGGTGTTAGATGAAGCTGACCGGATGTTAGATATGGGGTTTGAACCTCAGATACGAAGAATAGTTGAACAAGACACTATGCCTCCAAAAGGTGTCCGCCACACTATGATGTTTAGTGCTACTTTTCCTAAGGAAATACAGATGCTGGCCCGTGATTTCTTAGATGAGTACATATTTCTGGCTGTAGGAAGAGTTGGGTCTACTTCAGAGAACATCACACAAAAAGTGGTTTGGGTGGAGGAGATAGACAAAAGGTCATTTCTGCTTGACCTTCTAAATGCAACAGGCAAGGATTCCCTGACTCTAGTGTTTGTGGAGACCAAAAAGGGGGCAGATTCGCTGGAGGATTTCTTATACCATGAAGGATATGCTTGTACCAGTATCCATGGAGACCGTTCTCAGAGAGATAGGGAAGAGGCCCTTCACCAGTTCCGCTCAGGAAAAAGCCCAATTCTAGTGGCTACAGCAGTAGCAGCAAGAGGACTGGATATTTCAAATGTGAAGCATGTTATTAATTTTGACCTGCCTAGTGATATCGAAGAATATGTGCATCGCATAGGCCGTACAGGCCGTGTGGGAAACCTTGGTCTTGCCACCTCATTCTTTAATGAAAGGAATATAAATATCACAAAGGATTTACTGGATCTTCTTGTTGAAGCAAAACAAGAAGTGCCTTCTTGGTTAGAGAACATGGCTTTTGAACACCACTACAAGGGTAGCAGTCGTGGACGTTCTAAGAGCAGTCGATTTAGTGGAGGGTTTGGTGCCAGAGACTACCGACAGAGTAGCGGTGCCAGCAGTTCCAGCTTCAGCAGCAGCCGTGCAAGCAGCAGTCGAAGTGGTGGAGGTGGCCATGGCGGCAGTCGAGGATTTGGTGGAGGTGGCTACGGAGGCTTTTACAACAGTGATGGATATGGAGGGAATTATAACTCCCAGGGGGTTGACTGGTGGGGTAACCTCGAGTCTAGAGGGCCCTTCGAACAAAAACTCATCTCAGAAGAGGATCTGTGAGTTTAAACCCGCTGATCAGCCTCGACTGTGCCTTCTAGTTGCCAGCCATCTGTTGTTTGCCCCTCCCCCGTGCCTTCCTTGACCCTGGAAGGTGCCACTCCCACTGTCCTTTCCTAATAAAATGAGGAAATTGCATCGCATTGTCTGAGTAGGTGTCATTCTATTCTGGGGGGTGGGGTGGGGCAGGACAGCAAGGGGGAGGATTGGGAAGACAATAGCAGGCATGCTGGGGATGCGGTGGGCTCTATGGCTTCTGAGGCGGAAAGAACCAGCTGGGGCTCTAGGGGGTATCCCCACGCGCCCTGTAGCGGCGCATTAAGCGCGGCGGGTGTGGTGGTTACGCGCAGCGTGACCGCTACACTTGCCAGCGCCCTAGCGCCCGCTCCTTTCGCTTTCTTCCCTTCCTTTCTCGCCACGTTCGCCGGCTTTCCCCGTCAAGCTCTAAATCGGGGGCTCCCTTTAGGGTTCCGATTTAGTGCTTTACGGCACCTCGACCCCAAAAAACTTGATTAGGGTGATGGTTCACGTAGTGGGCCATCGCCCTGATAGACGGTTTTTCGCCCTTTGACGTTGGAGTCCACGTTCTTTAATAGTGGACTCTTGTTCCAAACTGGAACAACACTCAACCCTATCTCGGTCT |
