# Supplementary material for: Exosomal Lnc NEAT1 from endothelial cells promote bone regeneration by regulating macrophage polarization via DDX3X/NLRP3 axis
Source: J Nanobiotechnology. 2023 Mar 20;21:98. doi: 10.1186/s12951-023-01855-w (PMC10029245; doi:10.1186/s12951-023-01855-w)
Supplement: Supplementary file 5 — Additional file 5: Table S2. Primer sequences employed for reverse transcription-quantitative polymerase chain reaction. [file 12951_2023_1855_MOESM5_ESM.docx]

**Table S2.** Primer sequences employed for reverse transcription-quantitative polymerase chain reaction.

| **Gene** | **Forward primer（5'-3'）** | **Reverse primer（3'-5'）** |
| --- | --- | --- |
| **IL-6 Mouse** | **GGGACTGATGCTGGTGACAA** | **TCCACGATTTCCCAGAGAACA** |
| **IL-1β Mouse** | **CCCTGAACTCAACTGTGAAATAGCA** | **CCCAAGTCAAGGGCTTGGAA** |
| **IL-10 Mouse** | **GCCAGAGCCACATGCTCCTA** | **GATAAGGCTTGGCAACCCAAGTAA** |
| **Arg-1 Mouse** | **CTCCAAGCCAAAGTCCTTAGAG** | **GGAGCTGTCATTAGGGACATCA** |
| **β-actin Mouse** | **GTACGCCAACACAGTGCTG** | **CGTCATACTCCTGCTTGCTG** |
| **ALP Rat** | **CTGAACCGCAGGATGTGAA** | **GCCATCTTAGCAGCAACTTTC** |
| **OCN Rat** | **TCCGCTAGCTCGTCACAATTGG** | **CCTGACTGCATTCTGCCTCTCT** |
| **RUNX2 Rat** | **TTCGTCAGCGTCCTATCAGTTC** | **CTTCCATCAGCGTCAACACC** |
| **NEAT1**  **Human** | **GCC TTG TAG ATG GAG CTT GC** | **GCA CAA CAC AAT GAC ACC CT** |
| **GAPDH Rat** | **ACAGCAACAGGGTGGTGGAC** | **TTTGAGGGTGCAGCGAACTT** |
